# Supplementary material for: Using Natural Language Processing and Machine Learning to classify the status of kidney allograft in Electronic Medical Records written in Spanish
Source: PLoS One. 2025 May 8;20(5):e0322587. doi: 10.1371/journal.pone.0322587 (PMC12061128; doi:10.1371/journal.pone.0322587)
Supplement: S1 Preprocessing — (DOCX) [file pone.0322587.s001.docx]

**Supplementary Material**

Index

[Methods 1](#_Toc194054340)

[Table A.1. Keywords indicative of graft failure searched by text mining techniques 1](#_Toc194054341)

[Table A.2. Classification criteria of graft loss to obtain gold standard labels 2](#_Toc194054342)

[Table A.3. *Stopwords* removed from the clinical notes 3](#_Toc194054343)

[Table A.4. Medical abbreviations 3](#_Toc194054344)

[Table A.5. Definition of Spelling Errors in Medical Records 4](#_Toc194054345)

[Table A.6. Distribution of Spelling Errors in Medical Records 5](#_Toc194054346)

# Methods

Due to the high dimensionality of the data, a stringent p-value cutoff point of 0.01 was established to control for multiple testing and reduce the chance of false positives.

# Table A.1. Keywords indicative of graft failure searched by text mining techniques

| **Text mining search** | **English translation** |
| --- | --- |
| “Pérdida del injerto” | Graft loss |
| “Pérdida del trasplante” | Kidney loss |
| “Falla del trasplante” | Graft Failure |
| “Falla del injerto” | Transplant failure |
| “Nefrectomía” | Nephrectomy |
| “Exeresis” | Exeresis |
| “Trombosis” | Thrombosis |
| “Hemodiálisis” | Hemodialysis |
| “Diálisis” | Dialysis |

# Table A.2. Classification criteria of graft loss to obtain gold standard labels

| **Classification criteria** | **English translation** |
| --- | --- |
| Pérdida del injerto | Graft loss |
| Nefrectomía del injerto | Graft nephrectomy |
| Suspensión de terapia inmunosupresora de forma permanente | Permanent suspension of immunosuppressive therapy |
| Remisión a unidad de diálisis | Referral to dialysis unit |
| Hemodiálisis /diálisis peritoneal permanente | Permanent hemodialysis/peritoneal dialysis |
| Riñón no funcional | Non-functional kidney |
| Remisión a estudios pretrasplante | Referral to pretransplant studies |

# Table A.3. *Stopwords* removed from the clinical notes

**Stopwords**

| "de", "a", "al", "le", "las", "los", "y", "por", "se", "del", "la", "para", "que", "el" , "la", "del", "en", "ha", "nos", "pero", "si", "ya", "algo", "algunas", "alguna", "algunos", "esta", "está", "unos", "son", "con" "de", "a", "aa", "al", "le", "las", "los", "lo", "y", "por", "se", "del", "la", "para", "que", "el" , "la", "del", "en", "ha", "nos", "pero", "si", "ya", "algo", "algunas", "alguna", "algunos", "esta", "esto", "estos", "estan", "estar", "este", "cualquier", "unos", "son", "con", "hace", "es", "ahi", "aca", "asi", "cada", "cuando", "desde", "entonces", "esos", "esas", "fue", "fuimos", "ha", "hago", "me", "mis", "mi", "muy", "nos", "sra", "sr", "sus", "tal", "tales", "ti", "tiene", "mismo", "misma", "mismas", "mismos", "casi", "aun", "te", "tu", "yo", "ahora", "ante", "aqui", "bajo", "bien", "casi", "cerca", "ciertas", "cierta", "ciertos", "como", "consigue", "consigo", "da", "dan", "dice", "esa", "esas", "ese", "esos", "tenga", "tiene", "tener", "todos", "total" |
| --- |

**Custom stopwords**

| "refiere", "a", "dl", "aun", "enero", "febrero", "marzo", "abril", "mayo", "junio", "julio", "agosto", "septiembre", "octubre", "noviembre", "diciembre", "ademas", "presento", "presenta", "dra", "dr","dres", "ah", "luego", "mes", "meses", "dias", "dia", "horas", "hora", "minuto", "minutos", "am", "pm", "hoy", "mañana", "ayer", "mas", "menos", "mg", "miligramos", "microgramos", "microgramo", "decilitro", "mil", "miles", "mcg", "metros", "metro", "kilogramos", "kilogramo", "litros", "litro", "milimetros", "mililitro", "mililitros", "milimol", "milimoles", "min", "ml", "mkg", "mmol", "ms", "b", "c", "d", "e", "f", "g", "h", "i", "j", "k", "l", "m", "n","o", "p", "q", "r", "s", "t", "u", "v", "w", "x", "y", "z", "xl", "baez", "bernal", "bien", "bogota", "arauca", "chia", "sincelejo", "bolivar", "brasil", "buga", "buitrago", "burgos", "cada", "hace", "cali", "valle", "lili", "calera", "calima", "calle", "callejera", "camacho", "canada", "caprecom", "cafesalud", "carlos", "cartagena", "cartago", "casanare", "castaneda", "castaño", "castillo", "catalina", "cc", "ccd", "cd", "cesar","chia", "cienaga", "cm", "seg", "colombiana", "colombiana trasplantes" |
| --- |

# Table A.4. Medical abbreviations

Medical abbreviations were expanded into full words during the preprocessing of the free text

| **Abbreviation** | **Medical term** | **English translation** |
| --- | --- | --- |
| erc | enfermedad renal crónica | chronic kidney disease |
| dc | donante cadavérico | cadaveric donor |
| enf | enfermedad | disease |
| hta | hipertensión arterial | arterial hypertension |
| sec | secundario | secondary |
| dv | donante vivo | living donor |
| dvr | donante vivo relacionado | related living donor |
| qx | quirúrgico | surgical |
| h | horas | hours |
| tab | tableta | tablet |
| k | potasio | potassium |
| hg | hemoglobina | hemoglobin |
| hb | hemoglobina | hemoglobin |
| tfg | tasa de filtración glomerular | glomerular filtration rate |
| tto | tratamiento | treatment |
| tx | trasplante | transplant |
| cs | creatinina | creatinine |
| pop | posoperatorio | postoperative |
| acv | accidente cerebrovascular | cerebrovascular accident (stroke) |
| plaq | plaquetas | platelets |
| ta | tensión arterial | blood pressure |
| creat | creatinina | creatinine |
| rx | radiografía | X-ray |
| tgo | transaminasa | transaminase |
| tgp | transaminasa | transaminase |
| pte | paciente | patient |
| csa | ciclosporina | cyclosporine |
| dm | diabetes | diabetes |
| ch | cuadro hemático | blood count |
| po | parcial de orina | urinalysis |
| ab | antibiótico | antibiotic |

# Table A.5. Definition of Spelling Errors in Medical Records

| 1. **Accentuation Errors**: These occur when there are mistakes related to accents or diacritical marks. For example, writing “dia” instead of “día”. 2. **Omission Errors**: These happen when letters, words, or punctuation marks that should be present are missing. For example, writing “creatinina” as “creatnina”. 3. **Substitution Errors**: These involve replacing one letter, word, or punctuation mark with another incorrectly. For example, writing “hipertensión” as “hipertencsión”. 4. **Others**: This category includes any other types of errors that do not fit into the above categories. For example, errors due to incorrect word usage or formatting issues. |
| --- |

# Table A.6. Distribution of Spelling Errors in Medical Records

A total of 23009 words were analyzed, identifying a total of 13149 spelling errors.

| **Type of Error** | **Frequency** | **Percentage** |
| --- | --- | --- |
| Accentuation Errors | 1879 | 14.3% |
| Omission Errors | 8228 | 62.6% |
| Substitution Errors | 2272 | 17.3% |
| Others | 770 | 5.8% |
