# Supplementary material for: Using Natural Language Processing and Machine Learning to classify the status of kidney allograft in Electronic Medical Records written in Spanish
Source: PLoS One. 2025 May 8;20(5):e0322587. doi: 10.1371/journal.pone.0322587 (PMC12061128; doi:10.1371/journal.pone.0322587)
Supplement: S3 Model’s Performance — (HTML) [file pone.0322587.s003.html]

Logistic Regression, Random Forest and Neural networks models. Andrea Garcia


# Logistic Regression, Random Forest and Neural networks models. Andrea Garcia

# **Logistic Regression**

## **Data Partition- training, validation and test sets**

```
# Install and load the necessary libraries
#install.packages("ROSE")
#install.packages("caret")
library(ROSE)
library(caret)
library(glmnet)

# Check the distribution of the target variable
table(data.lasso$perdida_bin)
```

```
## 
##      0      1 
## 116503   1063
```

```
# Split the data into training (80%) and test (20%) sets
set.seed(69)
trainIndex <- createDataPartition(data.lasso$perdida_bin, p = 0.8, list = FALSE)
data.train <- data.lasso[trainIndex, ]
data.test <- data.lasso[-trainIndex, ]

# Check the distribution of the target variable in the test set
table(data.test$perdida_bin)
```

```
## 
##     0     1 
## 23296   217
```

```
# Apply undersampling to the training set to balance it 
data.train.balanced <- ovun.sample(perdida_bin ~ ., data = data.train, method = "under", seed = 69)$data

# Check the new distribution of the target variable in the balanced training set
table(data.train.balanced$perdida_bin)
```

```
## 
##   0   1 
## 834 846
```

# **Classifier 1-LR.Training and validation**

```
library(pROC)
library(ROCR)
# Perform 10-fold cross-validation on the balanced training set
set.seed(69)
folds <- createFolds(data.train.balanced$perdida_bin, k = 10)
accuracies <- numeric(10)
f1_scores <- numeric(10)
sensitivities <- numeric(10)
specificities <- numeric(10)
ppvs <- numeric(10)  # Positive Predictive Values
npvs <- numeric(10)  # Negative Predictive Values
aucs <- numeric(10)  # AUC values
models <- list()  # List to store the models of each fold
conf_matrices <- list()  # List to store the confusion matrices of each fold

for (i in 1:10) {
    # Segment your data by fold using the which() function 
    validationIndexes <- folds[[i]]
    validationData <- data.train.balanced[validationIndexes, ]
    trainData <- data.train.balanced[-validationIndexes, ]

    # Train the logistic regression model with L1 regularization (Lasso)
    x_train <- model.matrix(perdida_bin ~ nefrectomia + perdida + hemodialisi + unidad + terapia + dialisi, data = trainData)[, -1]
    y_train <- trainData$perdida_bin
    lasso_model <- cv.glmnet(x_train, y_train, family = "binomial", alpha = 1)
    
    # Predict on the validation data
    x_val <- model.matrix(perdida_bin ~ nefrectomia + perdida + hemodialisi + unidad + terapia + dialisi, data = validationData)[, -1]
    validation.pred <- predict(lasso_model, newx = x_val, s = "lambda.min", type = "response")
    
    # Adjust the decision threshold
    threshold <- 0.5  # You can try different values
    validation.pred <- ifelse(validation.pred > threshold, 1, 0)
    validation.pred <- factor(validation.pred, levels = c(1, 0))
    validation.actual <- factor(validationData$perdida_bin, levels = c(1, 0))

    # Compute the confusion matrix and extract the F1 score and sensitivity
    conf_matrix <- confusionMatrix(validation.pred, validation.actual)
    conf_matrices[[i]] <- conf_matrix  # Store the confusion matrix
    accuracies[i] <- conf_matrix$overall['Accuracy']
    f1_scores[i] <- conf_matrix$byClass['F1']
    sensitivities[i] <- conf_matrix$byClass['Sensitivity']
    specificities[i] <- conf_matrix$byClass['Specificity']
    ppvs[i] <- conf_matrix$byClass['Pos Pred Value']
    npvs[i] <- conf_matrix$byClass['Neg Pred Value']
    
    # Calculate AUC
    roc_obj <- roc(validation.actual, as.numeric(validation.pred))
    aucs[i] <- auc(roc_obj)
}

# Calculate the mean and standard deviation of the metrics
mean_f1 <- mean(f1_scores)
sd_f1 <- sd(f1_scores)
mean_sensitivity <- mean(sensitivities)
sd_sensitivity <- sd(sensitivities)
mean_specificity <- mean(specificities)
sd_specificity <- sd(specificities)
mean_ppv <- mean(ppvs)
sd_ppv <- sd(ppvs)
mean_npv <- mean(npvs)
sd_npv <- sd(npvs)
mean_accuracy <- mean(accuracies)
sd_accuracy <- sd(accuracies)
mean_auc <- mean(aucs)
sd_auc <- sd(aucs)

# Print the summarized results
cat("Mean F1 Score:", mean_f1, "\n")
```

```
## Mean F1 Score: 0.9525965
```

```
cat("Standard Deviation of F1 Score:", sd_f1, "\n")
```

```
## Standard Deviation of F1 Score: 0.01721805
```

```
cat("Mean Sensitivity:", mean_sensitivity, "\n")
```

```
## Mean Sensitivity: 0.9428112
```

```
cat("Standard Deviation of Sensitivity:", sd_sensitivity, "\n")
```

```
## Standard Deviation of Sensitivity: 0.02286775
```

```
cat("Mean Specificity:", mean_specificity, "\n")
```

```
## Mean Specificity: 0.9621485
```

```
cat("Standard Deviation of Specificity:", sd_specificity, "\n")
```

```
## Standard Deviation of Specificity: 0.01863148
```

```
cat("Mean PPV:", mean_ppv, "\n")
```

```
## Mean PPV: 0.9628049
```

```
cat("Standard Deviation of PPV:", sd_ppv, "\n")
```

```
## Standard Deviation of PPV: 0.01691357
```

```
cat("Mean NPV:", mean_npv, "\n")
```

```
## Mean NPV: 0.9414087
```

```
cat("Standard Deviation of NPV:", sd_npv, "\n")
```

```
## Standard Deviation of NPV: 0.02726829
```

```
cat("Mean Accuracy:", mean_accuracy, "\n")
```

```
## Mean Accuracy: 0.952381
```

```
cat("Standard Deviation of Accuracy:", sd_accuracy, "\n")
```

```
## Standard Deviation of Accuracy: 0.01861276
```

```
cat("Mean AUC:", mean_auc, "\n")
```

```
## Mean AUC: 0.9524798
```

```
cat("Standard Deviation of AUC:", sd_auc, "\n")
```

```
## Standard Deviation of AUC: 0.01821133
```

# **Testing classifier 1**

```
# Train the final model on the entire balanced training set
x_train_final <- model.matrix(perdida_bin ~ nefrectomia + perdida + hemodialisi + unidad + terapia + dialisi, data = data.train.balanced)[, -1]
y_train_final <- data.train.balanced$perdida_bin
final_model <- cv.glmnet(x_train_final, y_train_final, family = "binomial", alpha = 1)

# Evaluate the model on the test set
x_test <- model.matrix(perdida_bin ~ nefrectomia + perdida + hemodialisi + unidad + terapia + dialisi, data = data.test)[, -1]
test.pred <- predict(final_model, newx = x_test, s = "lambda.min", type = "response")
test.pred <- ifelse(test.pred > 0.9, 1, 0)  # Adjust the threshold as needed
test.pred <- factor(test.pred, levels = c(1, 0))
test.actual <- factor(data.test$perdida_bin, levels = c(1, 0))

# Compute the confusion matrix for the test set
test.confusion <- confusionMatrix(test.pred, test.actual)
print(test.confusion)
```

```
## Confusion Matrix and Statistics
## 
##           Reference
## Prediction     1     0
##          1   182   330
##          0    35 22966
##                                          
##                Accuracy : 0.9845         
##                  95% CI : (0.9828, 0.986)
##     No Information Rate : 0.9908         
##     P-Value [Acc > NIR] : 1              
##                                          
##                   Kappa : 0.4927         
##                                          
##  Mcnemar's Test P-Value : <2e-16         
##                                          
##             Sensitivity : 0.838710       
##             Specificity : 0.985834       
##          Pos Pred Value : 0.355469       
##          Neg Pred Value : 0.998478       
##              Prevalence : 0.009229       
##          Detection Rate : 0.007740       
##    Detection Prevalence : 0.021775       
##       Balanced Accuracy : 0.912272       
##                                          
##        'Positive' Class : 1              
##
```

```
# Calculate AUC
roc_obj <- roc(test.actual, as.numeric(test.pred))
```

```
## Setting levels: control = 1, case = 0
```

```
## Setting direction: controls < cases
```

```
auc_value <- auc(roc_obj)
cat("AUC:", auc_value, "\n")
```

```
## AUC: 0.9122721
```

# **Confusion matrix Classifier 1**

```
#Confusion matrix classifier 1
draw_confusion_matrix(test.confusion)
```

## **Random Forest**

## **Data Partition- training, validation and test sets**

```
library(ROSE)
library(caret)
library(glmnet)
# Check the distribution of the target variable
table(data.lasso$perdida_bin)
```

```
## 
##      0      1 
## 116503   1063
```

```
# Split the data into training (80%) and test (30%) sets
set.seed(69)
trainIndex <- createDataPartition(data.lasso$perdida_bin, p = 0.8, list = FALSE)
data.train <- data.lasso[trainIndex, ]
data.test <- data.lasso[-trainIndex, ]

# Check the distribution of the target variable in the test set
table(data.test$perdida_bin)
```

```
## 
##     0     1 
## 23296   217
```

```
# Apply undersampling to the training set to balance it 
data.train.balanced <- ovun.sample(perdida_bin ~ ., data = data.train, method = "under", seed = 69)$data

# Check the new distribution of the target variable in the balanced training set
table(data.train.balanced$perdida_bin)
```

```
## 
##   0   1 
## 834 846
```

```
# Convert the 'perdida_bin' column to a factor with levels 1 and 0 in the balanced, train, and test datasets
data.train.balanced$perdida_bin <- factor(data.train.balanced$perdida_bin, levels = c(1, 0))
data.train$perdida_bin <- factor(data.train$perdida_bin, levels = c(1, 0))
data.test$perdida_bin <- factor(data.test$perdida_bin, levels = c(1, 0))

# Ensure the balanced and train datasets are data frames
data.train.balanced <- as.data.frame(data.train.balanced)
data.train <- as.data.frame(data.train)
data.test <- as.data.frame(data.test)
```

# **Classifier 2-RF Training and validation**

```
library(caret)
library(randomForestSRC)
```

```
## 
##  randomForestSRC 3.3.1 
##  
##  Type rfsrc.news() to see new features, changes, and bug fixes. 
##
```

```
library(data.table)  # To create the comparison table

# Perform 10-fold cross-validation on the balanced training set
set.seed(69)
folds <- createFolds(data.train.balanced$perdida_bin, k = 10)
accuracies <- numeric(10)
f1_scores <- numeric(10)
sensitivities <- numeric(10)
specificities <- numeric(10)
ppvs <- numeric(10)  # Positive Predictive Values
npvs <- numeric(10)  # Negative Predictive Values
models <- list()  # List to store the models of each fold
conf_matrices <- list()  # List to store the confusion matrices of each fold

for (i in 1:10) {
    # Segment the data by fold using the which() function
    validationIndexes <- folds[[i]]
    validationData <- data.train.balanced[validationIndexes, ]
    trainData <- data.train.balanced[-validationIndexes, ]

    # Train the Random Forest model
    set.seed(69)
    rf_model <- rfsrc(perdida_bin ~ nefrectomia + perdida + hemodialisi + unidad + terapia + dialisi, 
                             data = trainData, 
                             ntree = 500,          # Number of trees
                             mtry = 2,             # Number of variables to consider at each split
                             nodesize = 10,        # Minimum size of terminal nodes
                             importance = TRUE,    # Calculate variable importance
                             sampsize = 0.9 * nrow(trainData))  # Sample size for each tree
    
    # Store the model
    models[[i]] <- rf_model
    
    # Predict on the validation data
    predicted <- predict(rf_model, validationData, type = "response")
    
    # Extract the predicted probabilities for the default class (assuming "1" is the default class)
    predicted_prob <- predicted$predicted[, "1"]
    
    # Convert probabilities to binary predictions using a threshold of 0.5
    predicted_prob2 <- ifelse(predicted_prob > 0.9, 1, 0)
    predicted_prob2 <- factor(predicted_prob2, levels = c(1, 0))
    validation.actual <- factor(validationData$perdida_bin, levels = c(1, 0))
    
    # Calculate the confusion matrix and extract metrics
    conf_matrix <- confusionMatrix(predicted_prob2, validation.actual)
    conf_matrices[[i]] <- conf_matrix  # Store the confusion matrix
    accuracies[i] <- conf_matrix$overall['Accuracy']
    f1_scores[i] <- conf_matrix$byClass['F1']
    sensitivities[i] <- conf_matrix$byClass['Sensitivity']
    specificities[i] <- conf_matrix$byClass['Specificity']
    ppvs[i] <- conf_matrix$byClass['Pos Pred Value']
    npvs[i] <- conf_matrix$byClass['Neg Pred Value']
}

# Create a data table with the results
results_table <- data.table(
  Fold = 1:10,
  Accuracy = accuracies,
  F1_Score = f1_scores,
  Sensitivity = sensitivities,
  Specificity = specificities,
  PPV = ppvs,
  NPV = npvs
)

# Print the results table
print(results_table)
```

```
##      Fold  Accuracy  F1_Score Sensitivity Specificity       PPV       NPV
##     <int>     <num>     <num>       <num>       <num>     <num>     <num>
##  1:     1 0.8639053 0.8456376   0.7411765   0.9880952 0.9843750 0.7904762
##  2:     2 0.9112426 0.9032258   0.8235294   1.0000000 1.0000000 0.8484848
##  3:     3 0.9161677 0.9090909   0.8333333   1.0000000 1.0000000 0.8556701
##  4:     4 0.8682635 0.8513514   0.7500000   0.9879518 0.9843750 0.7961165
##  5:     5 0.8750000 0.8571429   0.7500000   1.0000000 1.0000000 0.8000000
##  6:     6 0.8862275 0.8741722   0.7857143   0.9879518 0.9850746 0.8200000
##  7:     7 0.8869048 0.8741722   0.7764706   1.0000000 1.0000000 0.8137255
##  8:     8 0.8934911 0.8815789   0.7882353   1.0000000 1.0000000 0.8235294
##  9:     9 0.9166667 0.9113924   0.8470588   0.9879518 0.9863014 0.8631579
## 10:    10 0.9166667 0.9102564   0.8352941   1.0000000 1.0000000 0.8556701
```

```
# Calculate the mean and standard deviation of the metrics
mean_f1 <- mean(f1_scores)
sd_f1 <- sd(f1_scores)
mean_sensitivity <- mean(sensitivities)
sd_sensitivity <- sd(sensitivities)
mean_specificity <- mean(specificities)
sd_specificity <- sd(specificities)
mean_ppv <- mean(ppvs)
sd_ppv <- sd(ppvs)
mean_npv <- mean(npvs)
sd_npv <- sd(npvs)
mean_accuracy <- mean(accuracies)
sd_accuracy <- sd(accuracies)

# Print the summarized results
cat("Mean F1 Score:", mean_f1, "\n")
```

```
## Mean F1 Score: 0.8818021
```

```
cat("Standard Deviation of F1 Score:", sd_f1, "\n")
```

```
## Standard Deviation of F1 Score: 0.02549198
```

```
cat("Mean Sensitivity:", mean_sensitivity, "\n")
```

```
## Mean Sensitivity: 0.7930812
```

```
cat("Standard Deviation of Sensitivity:", sd_sensitivity, "\n")
```

```
## Standard Deviation of Sensitivity: 0.03944418
```

```
cat("Mean Specificity:", mean_specificity, "\n")
```

```
## Mean Specificity: 0.9951951
```

```
cat("Standard Deviation of Specificity:", sd_specificity, "\n")
```

```
## Standard Deviation of Specificity: 0.006203281
```

```
cat("Mean PPV:", mean_ppv, "\n")
```

```
## Mean PPV: 0.9940126
```

```
cat("Standard Deviation of PPV:", sd_ppv, "\n")
```

```
## Standard Deviation of PPV: 0.007747479
```

```
cat("Mean NPV:", mean_npv, "\n")
```

```
## Mean NPV: 0.8266831
```

```
cat("Standard Deviation of NPV:", sd_npv, "\n")
```

```
## Standard Deviation of NPV: 0.02721514
```

```
cat("Mean Accuracy:", mean_accuracy, "\n")
```

```
## Mean Accuracy: 0.8934536
```

```
cat("Standard Deviation of Accuracy:", sd_accuracy, "\n")
```

```
## Standard Deviation of Accuracy: 0.02069179
```

```
# Save the models, confusion matrices, and results table
save(models, file = "models.RData")
save(conf_matrices, file = "conf_matrices.RData")
save(results_table, file = "results_table.RData")
```

# **Testing Classifier 2- RF**

```
library(caret)
library(randomForest)
library(pROC)
library(ROCR)

# Load the model from the file
load("models.RData")

# Select the best model (for example, the seventh model)
best_model <- models[[9]]

# Evaluate the model on a new test set
new_test_data <- data.test
new_test_pred <- predict(best_model, new_test_data, type = "response")

# Extract the predicted probabilities for the default class (assuming "1" is the default class)
new_test_predicted_prob <- new_test_pred$predicted[, "1"]

# Convert the predictions to factors
new_test_predicted_prob2 <- ifelse(new_test_predicted_prob > 0.9, 1, 0)
new_test_predicted_prob2 <- factor(new_test_predicted_prob2, levels = c(1, 0))
new_test_actual <- factor(new_test_data$perdida_bin, levels = c(1, 0))

# Calculate the confusion matrix for the new test set
new_test_confusion <- confusionMatrix(new_test_predicted_prob2, new_test_actual)
print(new_test_confusion)
```

```
## Confusion Matrix and Statistics
## 
##           Reference
## Prediction     1     0
##          1   171   182
##          0    46 23114
##                                          
##                Accuracy : 0.9903         
##                  95% CI : (0.989, 0.9915)
##     No Information Rate : 0.9908         
##     P-Value [Acc > NIR] : 0.7848         
##                                          
##                   Kappa : 0.5954         
##                                          
##  Mcnemar's Test P-Value : <2e-16         
##                                          
##             Sensitivity : 0.788018       
##             Specificity : 0.992188       
##          Pos Pred Value : 0.484419       
##          Neg Pred Value : 0.998014       
##              Prevalence : 0.009229       
##          Detection Rate : 0.007273       
##    Detection Prevalence : 0.015013       
##       Balanced Accuracy : 0.890103       
##                                          
##        'Positive' Class : 1              
##
```

```
# Calculate the AUC
new_auc_value <- auc(new_test_actual, new_test_predicted_prob)
cat("AUC:", new_auc_value, "\n")
```

```
## AUC: 0.9816991
```

# **Confusion matrix Classifier 2**

```
#Confusion matrix 
draw_confusion_matrix(new_test_confusion)
```

# **Classifier 3-RF. Training and validation**

```
library(caret)
library(randomForestSRC)
library(data.table)  # To create the comparison table

# Perform 10-fold cross-validation on the balanced training set
set.seed(69)
folds <- createFolds(data.train.balanced$perdida_bin, k = 10)
accuracies <- numeric(10)
f1_scores <- numeric(10)
sensitivities <- numeric(10)
specificities <- numeric(10)
ppvs <- numeric(10)  # Positive Predictive Values
npvs <- numeric(10)  # Negative Predictive Values
models <- list()  # List to store the models of each fold
conf_matrices <- list()  # List to store the confusion matrices of each fold

for (i in 1:10) {
    # Segment the data by fold using the which() function
    validationIndexes <- folds[[i]]
    validationData <- data.train.balanced[validationIndexes, ]
    trainData <- data.train.balanced[-validationIndexes, ]

    # Train the Random Forest model
    set.seed(69)
    rf_model <- rfsrc(perdida_bin ~ nefrectomia + perdida + hemodialisi + unidad + terapia + dialisi, 
                             data = trainData, 
                             ntree = 500,          # Number of trees
                             mtry = 2,             # Number of variables to consider at each split
                             nodesize = 10,        # Minimum size of terminal nodes
                             importance = TRUE,    # Calculate variable importance
                             sampsize = 0.1 * nrow(trainData))  # Sample size for each tree
    
    # Store the model
    models[[i]] <- rf_model
    
    # Predict on the validation data
    predicted <- predict(rf_model, validationData, type = "response")
    
    # Extract the predicted probabilities for the default class (assuming "1" is the default class)
    predicted_prob <- predicted$predicted[, "1"]
    
    # Convert probabilities to binary predictions using a threshold of 0.5
    predicted_prob2 <- ifelse(predicted_prob > 0.9, 1, 0)
    predicted_prob2 <- factor(predicted_prob2, levels = c(1, 0))
    validation.actual <- factor(validationData$perdida_bin, levels = c(1, 0))
    
    # Calculate the confusion matrix and extract metrics
    conf_matrix <- confusionMatrix(predicted_prob2, validation.actual)
    conf_matrices[[i]] <- conf_matrix  # Store the confusion matrix
    accuracies[i] <- conf_matrix$overall['Accuracy']
    f1_scores[i] <- conf_matrix$byClass['F1']
    sensitivities[i] <- conf_matrix$byClass['Sensitivity']
    specificities[i] <- conf_matrix$byClass['Specificity']
    ppvs[i] <- conf_matrix$byClass['Pos Pred Value']
    npvs[i] <- conf_matrix$byClass['Neg Pred Value']
}

# Create a data table with the results
results_table <- data.table(
  Fold = 1:10,
  Accuracy = accuracies,
  F1_Score = f1_scores,
  Sensitivity = sensitivities,
  Specificity = specificities,
  PPV = ppvs,
  NPV = npvs
)

# Print the results table
print(results_table)
```

```
##      Fold  Accuracy  F1_Score Sensitivity Specificity       PPV       NPV
##     <int>     <num>     <num>       <num>       <num>     <num>     <num>
##  1:     1 0.8461538 0.8219178   0.7058824   0.9880952 0.9836066 0.7685185
##  2:     2 0.8698225 0.8513514   0.7411765   1.0000000 1.0000000 0.7924528
##  3:     3 0.8502994 0.8251748   0.7023810   1.0000000 1.0000000 0.7685185
##  4:     4 0.8383234 0.8111888   0.6904762   0.9879518 0.9830508 0.7592593
##  5:     5 0.8333333 0.8000000   0.6666667   1.0000000 1.0000000 0.7500000
##  6:     6 0.8622754 0.8435374   0.7380952   0.9879518 0.9841270 0.7884615
##  7:     7 0.8571429 0.8356164   0.7176471   1.0000000 1.0000000 0.7757009
##  8:     8 0.8402367 0.8111888   0.6823529   1.0000000 1.0000000 0.7567568
##  9:     9 0.8452381 0.8219178   0.7058824   0.9879518 0.9836066 0.7663551
## 10:    10 0.8750000 0.8590604   0.7529412   1.0000000 1.0000000 0.7980769
```

```
# Calculate the mean and standard deviation of the metrics
mean_f1 <- mean(f1_scores)
sd_f1 <- sd(f1_scores)
mean_sensitivity <- mean(sensitivities)
sd_sensitivity <- sd(sensitivities)
mean_specificity <- mean(specificities)
sd_specificity <- sd(specificities)
mean_ppv <- mean(ppvs)
sd_ppv <- sd(ppvs)
mean_npv <- mean(npvs)
sd_npv <- sd(npvs)
mean_accuracy <- mean(accuracies)
sd_accuracy <- sd(accuracies)

# Print the summarized results
cat("Mean F1 Score:", mean_f1, "\n")
```

```
## Mean F1 Score: 0.8280954
```

```
cat("Standard Deviation of F1 Score:", sd_f1, "\n")
```

```
## Standard Deviation of F1 Score: 0.01897532
```

```
cat("Mean Sensitivity:", mean_sensitivity, "\n")
```

```
## Mean Sensitivity: 0.7103501
```

```
cat("Standard Deviation of Sensitivity:", sd_sensitivity, "\n")
```

```
## Standard Deviation of Sensitivity: 0.02744244
```

```
cat("Mean Specificity:", mean_specificity, "\n")
```

```
## Mean Specificity: 0.9951951
```

```
cat("Standard Deviation of Specificity:", sd_specificity, "\n")
```

```
## Standard Deviation of Specificity: 0.006203281
```

```
cat("Mean PPV:", mean_ppv, "\n")
```

```
## Mean PPV: 0.9934391
```

```
cat("Standard Deviation of PPV:", sd_ppv, "\n")
```

```
## Standard Deviation of PPV: 0.008473892
```

```
cat("Mean NPV:", mean_npv, "\n")
```

```
## Mean NPV: 0.77241
```

```
cat("Standard Deviation of NPV:", sd_npv, "\n")
```

```
## Standard Deviation of NPV: 0.016042
```

```
cat("Mean Accuracy:", mean_accuracy, "\n")
```

```
## Mean Accuracy: 0.8517826
```

```
cat("Standard Deviation of Accuracy:", sd_accuracy, "\n")
```

```
## Standard Deviation of Accuracy: 0.01388391
```

```
# Save the models, confusion matrices, and results table
save(models, file = "models.RData")
save(conf_matrices, file = "conf_matrices.RData")
save(results_table, file = "results_table.RData")
```

# **Testing Classifier 3- RF**

```
library(caret)
library(randomForest)
library(pROC)
library(ROCR)

# Load the model from the file
load("models.RData")

# Select the best model (for example, the seventh model)
best_model <- models[[6]]

# Evaluate the model on a new test set
new_test_data <- data.test
new_test_pred <- predict(best_model, new_test_data, type = "response")

# Extract the predicted probabilities for the default class (assuming "1" is the default class)
new_test_predicted_prob <- new_test_pred$predicted[, "1"]

# Convert the predictions to factors
new_test_predicted_prob2 <- ifelse(new_test_predicted_prob > 0.9, 1, 0)
new_test_predicted_prob2 <- factor(new_test_predicted_prob2, levels = c(1, 0))
new_test_actual <- factor(new_test_data$perdida_bin, levels = c(1, 0))

# Calculate the confusion matrix for the new test set
new_test_confusion <- confusionMatrix(new_test_predicted_prob2, new_test_actual)
print(new_test_confusion)
```

```
## Confusion Matrix and Statistics
## 
##           Reference
## Prediction     1     0
##          1   156   106
##          0    61 23190
##                                           
##                Accuracy : 0.9929          
##                  95% CI : (0.9917, 0.9939)
##     No Information Rate : 0.9908          
##     P-Value [Acc > NIR] : 0.0002280       
##                                           
##                   Kappa : 0.6478          
##                                           
##  Mcnemar's Test P-Value : 0.0006621       
##                                           
##             Sensitivity : 0.718894        
##             Specificity : 0.995450        
##          Pos Pred Value : 0.595420        
##          Neg Pred Value : 0.997376        
##              Prevalence : 0.009229        
##          Detection Rate : 0.006635        
##    Detection Prevalence : 0.011143        
##       Balanced Accuracy : 0.857172        
##                                           
##        'Positive' Class : 1               
##
```

```
# Calculate the AUC
new_auc_value <- auc(new_test_actual, new_test_predicted_prob)
cat("AUC:", new_auc_value, "\n")
```

```
## AUC: 0.9833434
```

# **Confusion matrix Classifier 3**

```
#Confusion matrix 
draw_confusion_matrix(new_test_confusion)
```

# **Classifier 4-RF. Training and validation**

```
library(caret)
library(randomForestSRC)
library(data.table)  # To create the comparison table

# Perform 10-fold cross-validation on the balanced training set
set.seed(69)
folds <- createFolds(data.train.balanced$perdida_bin, k = 10)
accuracies <- numeric(10)
f1_scores <- numeric(10)
sensitivities <- numeric(10)
specificities <- numeric(10)
ppvs <- numeric(10)  # Positive Predictive Values
npvs <- numeric(10)  # Negative Predictive Values
models <- list()  # List to store the models of each fold
conf_matrices <- list()  # List to store the confusion matrices of each fold

for (i in 1:10) {
    # Segment the data by fold using the which() function
    validationIndexes <- folds[[i]]
    validationData <- data.train.balanced[validationIndexes, ]
    trainData <- data.train.balanced[-validationIndexes, ]

    # Train the Random Forest model
    set.seed(69)
    rf_model <- rfsrc(perdida_bin ~ nefrectomia + perdida + hemodialisi + unidad + terapia + dialisi, 
                             data = trainData, 
                             ntree = 500,          # Number of trees
                             mtry = 2,             # Number of variables to consider at each split
                             nodesize = 30,        # Minimum size of terminal nodes
                             importance = TRUE,    # Calculate variable importance
                             sampsize = 0.1 * nrow(trainData))  # Sample size for each tree
    
    # Store the model
    models[[i]] <- rf_model
    
    # Predict on the validation data
    predicted <- predict(rf_model, validationData, type = "response")
    
    # Extract the predicted probabilities for the default class (assuming "1" is the default class)
    predicted_prob <- predicted$predicted[, "1"]
    
    # Convert probabilities to binary predictions using a threshold of 0.5
    predicted_prob2 <- ifelse(predicted_prob > 0.9, 1, 0)
    predicted_prob2 <- factor(predicted_prob2, levels = c(1, 0))
    validation.actual <- factor(validationData$perdida_bin, levels = c(1, 0))
    
    # Calculate the confusion matrix and extract metrics
    conf_matrix <- confusionMatrix(predicted_prob2, validation.actual)
    conf_matrices[[i]] <- conf_matrix  # Store the confusion matrix
    accuracies[i] <- conf_matrix$overall['Accuracy']
    f1_scores[i] <- conf_matrix$byClass['F1']
    sensitivities[i] <- conf_matrix$byClass['Sensitivity']
    specificities[i] <- conf_matrix$byClass['Specificity']
    ppvs[i] <- conf_matrix$byClass['Pos Pred Value']
    npvs[i] <- conf_matrix$byClass['Neg Pred Value']
}

# Create a data table with the results
results_table <- data.table(
  Fold = 1:10,
  Accuracy = accuracies,
  F1_Score = f1_scores,
  Sensitivity = sensitivities,
  Specificity = specificities,
  PPV = ppvs,
  NPV = npvs
)

# Print the results table
print(results_table)
```

```
##      Fold  Accuracy  F1_Score Sensitivity Specificity       PPV       NPV
##     <int>     <num>     <num>       <num>       <num>     <num>     <num>
##  1:     1 0.8461538 0.8219178   0.7058824   0.9880952 0.9836066 0.7685185
##  2:     2 0.8639053 0.8435374   0.7294118   1.0000000 1.0000000 0.7850467
##  3:     3 0.8502994 0.8251748   0.7023810   1.0000000 1.0000000 0.7685185
##  4:     4 0.8383234 0.8111888   0.6904762   0.9879518 0.9830508 0.7592593
##  5:     5 0.8333333 0.8000000   0.6666667   1.0000000 1.0000000 0.7500000
##  6:     6 0.8622754 0.8435374   0.7380952   0.9879518 0.9841270 0.7884615
##  7:     7 0.8571429 0.8356164   0.7176471   1.0000000 1.0000000 0.7757009
##  8:     8 0.8520710 0.8275862   0.7058824   1.0000000 1.0000000 0.7706422
##  9:     9 0.8452381 0.8219178   0.7058824   0.9879518 0.9836066 0.7663551
## 10:    10 0.8809524 0.8666667   0.7647059   1.0000000 1.0000000 0.8058252
```

```
# Calculate the mean and standard deviation of the metrics
mean_f1 <- mean(f1_scores)
sd_f1 <- sd(f1_scores)
mean_sensitivity <- mean(sensitivities)
sd_sensitivity <- sd(sensitivities)
mean_specificity <- mean(specificities)
sd_specificity <- sd(specificities)
mean_ppv <- mean(ppvs)
sd_ppv <- sd(ppvs)
mean_npv <- mean(npvs)
sd_npv <- sd(npvs)
mean_accuracy <- mean(accuracies)
sd_accuracy <- sd(accuracies)

# Print the summarized results
cat("Mean F1 Score:", mean_f1, "\n")
```

```
## Mean F1 Score: 0.8297143
```

```
cat("Standard Deviation of F1 Score:", sd_f1, "\n")
```

```
## Standard Deviation of F1 Score: 0.01872405
```

```
cat("Mean Sensitivity:", mean_sensitivity, "\n")
```

```
## Mean Sensitivity: 0.7127031
```

```
cat("Standard Deviation of Sensitivity:", sd_sensitivity, "\n")
```

```
## Standard Deviation of Sensitivity: 0.02689935
```

```
cat("Mean Specificity:", mean_specificity, "\n")
```

```
## Mean Specificity: 0.9951951
```

```
cat("Standard Deviation of Specificity:", sd_specificity, "\n")
```

```
## Standard Deviation of Specificity: 0.006203281
```

```
cat("Mean PPV:", mean_ppv, "\n")
```

```
## Mean PPV: 0.9934391
```

```
cat("Standard Deviation of PPV:", sd_ppv, "\n")
```

```
## Standard Deviation of PPV: 0.008473892
```

```
cat("Mean NPV:", mean_npv, "\n")
```

```
## Mean NPV: 0.7738328
```

```
cat("Standard Deviation of NPV:", sd_npv, "\n")
```

```
## Standard Deviation of NPV: 0.01588055
```

```
cat("Mean Accuracy:", mean_accuracy, "\n")
```

```
## Mean Accuracy: 0.8529695
```

```
cat("Standard Deviation of Accuracy:", sd_accuracy, "\n")
```

```
## Standard Deviation of Accuracy: 0.01382793
```

```
# Save the models, confusion matrices, and results table
save(models, file = "models.RData")
save(conf_matrices, file = "conf_matrices.RData")
save(results_table, file = "results_table.RData")
```

# **Testing Classifier 4- RF**

```
library(caret)
library(randomForest)
library(pROC)
library(ROCR)

# Load the model from the file
load("models.RData")

# Select the best model (for example, the seventh model)
best_model <- models[[10]]

# Evaluate the model on a new test set
new_test_data <- data.test
new_test_pred <- predict(best_model, new_test_data, type = "response")

# Extract the predicted probabilities for the default class (assuming "1" is the default class)
new_test_predicted_prob <- new_test_pred$predicted[, "1"]

# Convert the predictions to factors
new_test_predicted_prob2 <- ifelse(new_test_predicted_prob > 0.9, 1, 0)
new_test_predicted_prob2 <- factor(new_test_predicted_prob2, levels = c(1, 0))
new_test_actual <- factor(new_test_data$perdida_bin, levels = c(1, 0))

# Calculate the confusion matrix for the new test set
new_test_confusion <- confusionMatrix(new_test_predicted_prob2, new_test_actual)
print(new_test_confusion)
```

```
## Confusion Matrix and Statistics
## 
##           Reference
## Prediction     1     0
##          1   156   108
##          0    61 23188
##                                           
##                Accuracy : 0.9928          
##                  95% CI : (0.9916, 0.9939)
##     No Information Rate : 0.9908          
##     P-Value [Acc > NIR] : 0.0003937       
##                                           
##                   Kappa : 0.6451          
##                                           
##  Mcnemar's Test P-Value : 0.0004025       
##                                           
##             Sensitivity : 0.718894        
##             Specificity : 0.995364        
##          Pos Pred Value : 0.590909        
##          Neg Pred Value : 0.997376        
##              Prevalence : 0.009229        
##          Detection Rate : 0.006635        
##    Detection Prevalence : 0.011228        
##       Balanced Accuracy : 0.857129        
##                                           
##        'Positive' Class : 1               
##
```

```
# Calculate the AUC
new_auc_value <- auc(new_test_actual, new_test_predicted_prob)
cat("AUC:", new_auc_value, "\n")
```

```
## AUC: 0.9824301
```

# **Confusion matrix Classifier 4**

```
#Confusion matrix 
draw_confusion_matrix(new_test_confusion)
```

# **Classifier 5-RF. Training and validation**

```
library(caret)
library(randomForestSRC)
library(data.table)  # To create the comparison table

# Perform 10-fold cross-validation on the balanced training set
set.seed(69)
folds <- createFolds(data.train.balanced$perdida_bin, k = 10)
accuracies <- numeric(10)
f1_scores <- numeric(10)
sensitivities <- numeric(10)
specificities <- numeric(10)
ppvs <- numeric(10)  # Positive Predictive Values
npvs <- numeric(10)  # Negative Predictive Values
models <- list()  # List to store the models of each fold
conf_matrices <- list()  # List to store the confusion matrices of each fold

for (i in 1:10) {
    # Segment the data by fold using the which() function
    validationIndexes <- folds[[i]]
    validationData <- data.train.balanced[validationIndexes, ]
    trainData <- data.train.balanced[-validationIndexes, ]

    # Train the Random Forest model
    set.seed(69)
    rf_model <- rfsrc(perdida_bin ~ nefrectomia + perdida + hemodialisi + unidad + terapia + dialisi, 
                             data = trainData, 
                             ntree = 500,          # Number of trees
                             mtry = 2,             # Number of variables to consider at each split
                             nodesize = 30,        # Minimum size of terminal nodes
                             importance = TRUE,    # Calculate variable importance
                             sampsize = 0.1 * nrow(trainData))  # Sample size for each tree
    
    # Store the model
    models[[i]] <- rf_model
    
    # Predict on the validation data
    predicted <- predict(rf_model, validationData, type = "response")
    
    # Extract the predicted probabilities for the default class (assuming "1" is the default class)
    predicted_prob <- predicted$predicted[, "1"]
    
    # Convert probabilities to binary predictions using a threshold of 0.5
    predicted_prob2 <- ifelse(predicted_prob > 0.9, 1, 0)
    predicted_prob2 <- factor(predicted_prob2, levels = c(1, 0))
    validation.actual <- factor(validationData$perdida_bin, levels = c(1, 0))
    
    # Calculate the confusion matrix and extract metrics
    conf_matrix <- confusionMatrix(predicted_prob2, validation.actual)
    conf_matrices[[i]] <- conf_matrix  # Store the confusion matrix
    accuracies[i] <- conf_matrix$overall['Accuracy']
    f1_scores[i] <- conf_matrix$byClass['F1']
    sensitivities[i] <- conf_matrix$byClass['Sensitivity']
    specificities[i] <- conf_matrix$byClass['Specificity']
    ppvs[i] <- conf_matrix$byClass['Pos Pred Value']
    npvs[i] <- conf_matrix$byClass['Neg Pred Value']
}

# Create a data table with the results
results_table <- data.table(
  Fold = 1:10,
  Accuracy = accuracies,
  F1_Score = f1_scores,
  Sensitivity = sensitivities,
  Specificity = specificities,
  PPV = ppvs,
  NPV = npvs
)

# Print the results table
print(results_table)
```

```
##      Fold  Accuracy  F1_Score Sensitivity Specificity       PPV       NPV
##     <int>     <num>     <num>       <num>       <num>     <num>     <num>
##  1:     1 0.8461538 0.8219178   0.7058824   0.9880952 0.9836066 0.7685185
##  2:     2 0.8639053 0.8435374   0.7294118   1.0000000 1.0000000 0.7850467
##  3:     3 0.8502994 0.8251748   0.7023810   1.0000000 1.0000000 0.7685185
##  4:     4 0.8383234 0.8111888   0.6904762   0.9879518 0.9830508 0.7592593
##  5:     5 0.8333333 0.8000000   0.6666667   1.0000000 1.0000000 0.7500000
##  6:     6 0.8622754 0.8435374   0.7380952   0.9879518 0.9841270 0.7884615
##  7:     7 0.8571429 0.8356164   0.7176471   1.0000000 1.0000000 0.7757009
##  8:     8 0.8520710 0.8275862   0.7058824   1.0000000 1.0000000 0.7706422
##  9:     9 0.8452381 0.8219178   0.7058824   0.9879518 0.9836066 0.7663551
## 10:    10 0.8809524 0.8666667   0.7647059   1.0000000 1.0000000 0.8058252
```

```
# Calculate the mean and standard deviation of the metrics
mean_f1 <- mean(f1_scores)
sd_f1 <- sd(f1_scores)
mean_sensitivity <- mean(sensitivities)
sd_sensitivity <- sd(sensitivities)
mean_specificity <- mean(specificities)
sd_specificity <- sd(specificities)
mean_ppv <- mean(ppvs)
sd_ppv <- sd(ppvs)
mean_npv <- mean(npvs)
sd_npv <- sd(npvs)
mean_accuracy <- mean(accuracies)
sd_accuracy <- sd(accuracies)

# Print the summarized results
cat("Mean F1 Score:", mean_f1, "\n")
```

```
## Mean F1 Score: 0.8297143
```

```
cat("Standard Deviation of F1 Score:", sd_f1, "\n")
```

```
## Standard Deviation of F1 Score: 0.01872405
```

```
cat("Mean Sensitivity:", mean_sensitivity, "\n")
```

```
## Mean Sensitivity: 0.7127031
```

```
cat("Standard Deviation of Sensitivity:", sd_sensitivity, "\n")
```

```
## Standard Deviation of Sensitivity: 0.02689935
```

```
cat("Mean Specificity:", mean_specificity, "\n")
```

```
## Mean Specificity: 0.9951951
```

```
cat("Standard Deviation of Specificity:", sd_specificity, "\n")
```

```
## Standard Deviation of Specificity: 0.006203281
```

```
cat("Mean PPV:", mean_ppv, "\n")
```

```
## Mean PPV: 0.9934391
```

```
cat("Standard Deviation of PPV:", sd_ppv, "\n")
```

```
## Standard Deviation of PPV: 0.008473892
```

```
cat("Mean NPV:", mean_npv, "\n")
```

```
## Mean NPV: 0.7738328
```

```
cat("Standard Deviation of NPV:", sd_npv, "\n")
```

```
## Standard Deviation of NPV: 0.01588055
```

```
cat("Mean Accuracy:", mean_accuracy, "\n")
```

```
## Mean Accuracy: 0.8529695
```

```
cat("Standard Deviation of Accuracy:", sd_accuracy, "\n")
```

```
## Standard Deviation of Accuracy: 0.01382793
```

```
# Save the models, confusion matrices, and results table
save(models, file = "models.RData")
save(conf_matrices, file = "conf_matrices.RData")
save(results_table, file = "results_table.RData")
```

# **Testing Classifier 5- RF**

```
library(caret)
library(randomForest)
library(pROC)
library(ROCR)

# Load the model from the file
load("models.RData")

# Select the best model (for example, the seventh model)
best_model <- models[[6]]

# Evaluate the model on a new test set
new_test_data <- data.test
new_test_pred <- predict(best_model, new_test_data, type = "response")

# Extract the predicted probabilities for the default class (assuming "1" is the default class)
new_test_predicted_prob <- new_test_pred$predicted[, "1"]

# Convert the predictions to factors
new_test_predicted_prob2 <- ifelse(new_test_predicted_prob > 0.8, 1, 0)
new_test_predicted_prob2 <- factor(new_test_predicted_prob2, levels = c(1, 0))
new_test_actual <- factor(new_test_data$perdida_bin, levels = c(1, 0))

# Calculate the confusion matrix for the new test set
new_test_confusion <- confusionMatrix(new_test_predicted_prob2, new_test_actual)
print(new_test_confusion)
```

```
## Confusion Matrix and Statistics
## 
##           Reference
## Prediction     1     0
##          1   181   279
##          0    36 23017
##                                          
##                Accuracy : 0.9866         
##                  95% CI : (0.9851, 0.988)
##     No Information Rate : 0.9908         
##     P-Value [Acc > NIR] : 1              
##                                          
##                   Kappa : 0.5288         
##                                          
##  Mcnemar's Test P-Value : <2e-16         
##                                          
##             Sensitivity : 0.834101       
##             Specificity : 0.988024       
##          Pos Pred Value : 0.393478       
##          Neg Pred Value : 0.998438       
##              Prevalence : 0.009229       
##          Detection Rate : 0.007698       
##    Detection Prevalence : 0.019564       
##       Balanced Accuracy : 0.911063       
##                                          
##        'Positive' Class : 1              
##
```

```
# Calculate the AUC
new_auc_value <- auc(new_test_actual, new_test_predicted_prob)
cat("AUC:", new_auc_value, "\n")
```

```
## AUC: 0.9833369
```

# **Confusion matrix Classifier 5**

```
#Confusion matrix 
draw_confusion_matrix(new_test_confusion)
```

# **Classifier 6-RF. Training and validation**

```
library(caret)
library(randomForestSRC)
library(data.table)  # To create the comparison table

# Perform 10-fold cross-validation on the balanced training set
set.seed(69)
folds <- createFolds(data.train.balanced$perdida_bin, k = 10)
accuracies <- numeric(10)
f1_scores <- numeric(10)
sensitivities <- numeric(10)
specificities <- numeric(10)
ppvs <- numeric(10)  # Positive Predictive Values
npvs <- numeric(10)  # Negative Predictive Values
models <- list()  # List to store the models of each fold
conf_matrices <- list()  # List to store the confusion matrices of each fold

for (i in 1:10) {
    # Segment the data by fold using the which() function
    validationIndexes <- folds[[i]]
    validationData <- data.train.balanced[validationIndexes, ]
    trainData <- data.train.balanced[-validationIndexes, ]

    # Train the Random Forest model
    set.seed(69)
    rf_model <- rfsrc(perdida_bin ~ nefrectomia + perdida + hemodialisi + unidad + terapia + dialisi, 
                             data = trainData, 
                             ntree = 500,          # Number of trees
                             mtry = 2,             # Number of variables to consider at each split
                             nodesize = 30,        # Minimum size of terminal nodes
                             importance = TRUE,    # Calculate variable importance
                             sampsize = 0.1 * nrow(trainData))  # Sample size for each tree
    
    # Store the model
    models[[i]] <- rf_model
    
    # Predict on the validation data
    predicted <- predict(rf_model, validationData, type = "response")
    
    # Extract the predicted probabilities for the default class (assuming "1" is the default class)
    predicted_prob <- predicted$predicted[, "1"]
    
    # Convert probabilities to binary predictions using a threshold of 0.5
    predicted_prob2 <- ifelse(predicted_prob > 0.9, 1, 0)
    predicted_prob2 <- factor(predicted_prob2, levels = c(1, 0))
    validation.actual <- factor(validationData$perdida_bin, levels = c(1, 0))
    
    # Calculate the confusion matrix and extract metrics
    conf_matrix <- confusionMatrix(predicted_prob2, validation.actual)
    conf_matrices[[i]] <- conf_matrix  # Store the confusion matrix
    accuracies[i] <- conf_matrix$overall['Accuracy']
    f1_scores[i] <- conf_matrix$byClass['F1']
    sensitivities[i] <- conf_matrix$byClass['Sensitivity']
    specificities[i] <- conf_matrix$byClass['Specificity']
    ppvs[i] <- conf_matrix$byClass['Pos Pred Value']
    npvs[i] <- conf_matrix$byClass['Neg Pred Value']
}

# Create a data table with the results
results_table <- data.table(
  Fold = 1:10,
  Accuracy = accuracies,
  F1_Score = f1_scores,
  Sensitivity = sensitivities,
  Specificity = specificities,
  PPV = ppvs,
  NPV = npvs
)

# Print the results table
print(results_table)
```

```
##      Fold  Accuracy  F1_Score Sensitivity Specificity       PPV       NPV
##     <int>     <num>     <num>       <num>       <num>     <num>     <num>
##  1:     1 0.8461538 0.8219178   0.7058824   0.9880952 0.9836066 0.7685185
##  2:     2 0.8639053 0.8435374   0.7294118   1.0000000 1.0000000 0.7850467
##  3:     3 0.8502994 0.8251748   0.7023810   1.0000000 1.0000000 0.7685185
##  4:     4 0.8383234 0.8111888   0.6904762   0.9879518 0.9830508 0.7592593
##  5:     5 0.8333333 0.8000000   0.6666667   1.0000000 1.0000000 0.7500000
##  6:     6 0.8622754 0.8435374   0.7380952   0.9879518 0.9841270 0.7884615
##  7:     7 0.8571429 0.8356164   0.7176471   1.0000000 1.0000000 0.7757009
##  8:     8 0.8520710 0.8275862   0.7058824   1.0000000 1.0000000 0.7706422
##  9:     9 0.8452381 0.8219178   0.7058824   0.9879518 0.9836066 0.7663551
## 10:    10 0.8809524 0.8666667   0.7647059   1.0000000 1.0000000 0.8058252
```

```
# Calculate the mean and standard deviation of the metrics
mean_f1 <- mean(f1_scores)
sd_f1 <- sd(f1_scores)
mean_sensitivity <- mean(sensitivities)
sd_sensitivity <- sd(sensitivities)
mean_specificity <- mean(specificities)
sd_specificity <- sd(specificities)
mean_ppv <- mean(ppvs)
sd_ppv <- sd(ppvs)
mean_npv <- mean(npvs)
sd_npv <- sd(npvs)
mean_accuracy <- mean(accuracies)
sd_accuracy <- sd(accuracies)

# Print the summarized results
cat("Mean F1 Score:", mean_f1, "\n")
```

```
## Mean F1 Score: 0.8297143
```

```
cat("Standard Deviation of F1 Score:", sd_f1, "\n")
```

```
## Standard Deviation of F1 Score: 0.01872405
```

```
cat("Mean Sensitivity:", mean_sensitivity, "\n")
```

```
## Mean Sensitivity: 0.7127031
```

```
cat("Standard Deviation of Sensitivity:", sd_sensitivity, "\n")
```

```
## Standard Deviation of Sensitivity: 0.02689935
```

```
cat("Mean Specificity:", mean_specificity, "\n")
```

```
## Mean Specificity: 0.9951951
```

```
cat("Standard Deviation of Specificity:", sd_specificity, "\n")
```

```
## Standard Deviation of Specificity: 0.006203281
```

```
cat("Mean PPV:", mean_ppv, "\n")
```

```
## Mean PPV: 0.9934391
```

```
cat("Standard Deviation of PPV:", sd_ppv, "\n")
```

```
## Standard Deviation of PPV: 0.008473892
```

```
cat("Mean NPV:", mean_npv, "\n")
```

```
## Mean NPV: 0.7738328
```

```
cat("Standard Deviation of NPV:", sd_npv, "\n")
```

```
## Standard Deviation of NPV: 0.01588055
```

```
cat("Mean Accuracy:", mean_accuracy, "\n")
```

```
## Mean Accuracy: 0.8529695
```

```
cat("Standard Deviation of Accuracy:", sd_accuracy, "\n")
```

```
## Standard Deviation of Accuracy: 0.01382793
```

```
# Save the models, confusion matrices, and results table
save(models, file = "models.RData")
save(conf_matrices, file = "conf_matrices.RData")
save(results_table, file = "results_table.RData")
```

# **Testing Classifier 6- RF**

```
library(caret)
library(randomForest)
library(pROC)
library(ROCR)

# Load the model from the file
load("models.RData")

# Select the best model (for example, the seventh model)
best_model <- models[[10]]

# Evaluate the model on a new test set
new_test_data <- data.test
new_test_pred <- predict(best_model, new_test_data, type = "response")

# Extract the predicted probabilities for the default class (assuming "1" is the default class)
new_test_predicted_prob <- new_test_pred$predicted[, "1"]

# Convert the predictions to factors
new_test_predicted_prob2 <- ifelse(new_test_predicted_prob > 0.9, 1, 0)
new_test_predicted_prob2 <- factor(new_test_predicted_prob2, levels = c(1, 0))
new_test_actual <- factor(new_test_data$perdida_bin, levels = c(1, 0))

# Calculate the confusion matrix for the new test set
new_test_confusion <- confusionMatrix(new_test_predicted_prob2, new_test_actual)
print(new_test_confusion)
```

```
## Confusion Matrix and Statistics
## 
##           Reference
## Prediction     1     0
##          1   156   108
##          0    61 23188
##                                           
##                Accuracy : 0.9928          
##                  95% CI : (0.9916, 0.9939)
##     No Information Rate : 0.9908          
##     P-Value [Acc > NIR] : 0.0003937       
##                                           
##                   Kappa : 0.6451          
##                                           
##  Mcnemar's Test P-Value : 0.0004025       
##                                           
##             Sensitivity : 0.718894        
##             Specificity : 0.995364        
##          Pos Pred Value : 0.590909        
##          Neg Pred Value : 0.997376        
##              Prevalence : 0.009229        
##          Detection Rate : 0.006635        
##    Detection Prevalence : 0.011228        
##       Balanced Accuracy : 0.857129        
##                                           
##        'Positive' Class : 1               
##
```

```
# Calculate the AUC
new_auc_value <- auc(new_test_actual, new_test_predicted_prob)
cat("AUC:", new_auc_value, "\n")
```

```
## AUC: 0.9824301
```

# **Confusion matrix Classifier 6**

```
#Confusion matrix 
draw_confusion_matrix(new_test_confusion)
```

# **Classifier 7-RF. Training and validation**

```
library(caret)
library(randomForestSRC)
library(data.table)  # To create the comparison table

# Perform 10-fold cross-validation on the balanced training set
set.seed(69)
folds <- createFolds(data.train.balanced$perdida_bin, k = 10)
accuracies <- numeric(10)
f1_scores <- numeric(10)
sensitivities <- numeric(10)
specificities <- numeric(10)
ppvs <- numeric(10)  # Positive Predictive Values
npvs <- numeric(10)  # Negative Predictive Values
models <- list()  # List to store the models of each fold
conf_matrices <- list()  # List to store the confusion matrices of each fold

for (i in 1:10) {
    # Segment the data by fold using the which() function
    validationIndexes <- folds[[i]]
    validationData <- data.train.balanced[validationIndexes, ]
    trainData <- data.train.balanced[-validationIndexes, ]

    # Train the Random Forest model
    set.seed(69)
    rf_model <- rfsrc(perdida_bin ~ nefrectomia + perdida + hemodialisi + unidad + terapia + dialisi, 
                             data = trainData, 
                             ntree = 500,          # Number of trees
                             mtry = 2,             # Number of variables to consider at each split
                             nodesize = 50,        # Minimum size of terminal nodes
                             importance = TRUE,    # Calculate variable importance
                             sampsize = 0.5 * nrow(trainData))  # Sample size for each tree
    
    # Store the model
    models[[i]] <- rf_model
    
    # Predict on the validation data
    predicted <- predict(rf_model, validationData, type = "response")
    
    # Extract the predicted probabilities for the default class (assuming "1" is the default class)
    predicted_prob <- predicted$predicted[, "1"]
    
    # Convert probabilities to binary predictions using a threshold of 0.5
    predicted_prob2 <- ifelse(predicted_prob > 0.9, 1, 0)
    predicted_prob2 <- factor(predicted_prob2, levels = c(1, 0))
    validation.actual <- factor(validationData$perdida_bin, levels = c(1, 0))
    
    # Calculate the confusion matrix and extract metrics
    conf_matrix <- confusionMatrix(predicted_prob2, validation.actual)
    conf_matrices[[i]] <- conf_matrix  # Store the confusion matrix
    accuracies[i] <- conf_matrix$overall['Accuracy']
    f1_scores[i] <- conf_matrix$byClass['F1']
    sensitivities[i] <- conf_matrix$byClass['Sensitivity']
    specificities[i] <- conf_matrix$byClass['Specificity']
    ppvs[i] <- conf_matrix$byClass['Pos Pred Value']
    npvs[i] <- conf_matrix$byClass['Neg Pred Value']
}

# Create a data table with the results
results_table <- data.table(
  Fold = 1:10,
  Accuracy = accuracies,
  F1_Score = f1_scores,
  Sensitivity = sensitivities,
  Specificity = specificities,
  PPV = ppvs,
  NPV = npvs
)

# Print the results table
print(results_table)
```

```
##      Fold  Accuracy  F1_Score Sensitivity Specificity       PPV       NPV
##     <int>     <num>     <num>       <num>       <num>     <num>     <num>
##  1:     1 0.8461538 0.8219178   0.7058824   0.9880952 0.9836066 0.7685185
##  2:     2 0.8579882 0.8356164   0.7176471   1.0000000 1.0000000 0.7777778
##  3:     3 0.8443114 0.8169014   0.6904762   1.0000000 1.0000000 0.7614679
##  4:     4 0.8323353 0.8028169   0.6785714   0.9879518 0.9827586 0.7522936
##  5:     5 0.8333333 0.8000000   0.6666667   1.0000000 1.0000000 0.7500000
##  6:     6 0.8622754 0.8435374   0.7380952   0.9879518 0.9841270 0.7884615
##  7:     7 0.8392857 0.8111888   0.6823529   1.0000000 1.0000000 0.7545455
##  8:     8 0.8639053 0.8435374   0.7294118   1.0000000 1.0000000 0.7850467
##  9:     9 0.8452381 0.8219178   0.7058824   0.9879518 0.9836066 0.7663551
## 10:    10 0.8630952 0.8435374   0.7294118   1.0000000 1.0000000 0.7830189
```

```
# Calculate the mean and standard deviation of the metrics
mean_f1 <- mean(f1_scores)
sd_f1 <- sd(f1_scores)
mean_sensitivity <- mean(sensitivities)
sd_sensitivity <- sd(sensitivities)
mean_specificity <- mean(specificities)
sd_specificity <- sd(specificities)
mean_ppv <- mean(ppvs)
sd_ppv <- sd(ppvs)
mean_npv <- mean(npvs)
sd_npv <- sd(npvs)
mean_accuracy <- mean(accuracies)
sd_accuracy <- sd(accuracies)

# Print the summarized results
cat("Mean F1 Score:", mean_f1, "\n")
```

```
## Mean F1 Score: 0.8240971
```

```
cat("Standard Deviation of F1 Score:", sd_f1, "\n")
```

```
## Standard Deviation of F1 Score: 0.01675938
```

```
cat("Mean Sensitivity:", mean_sensitivity, "\n")
```

```
## Mean Sensitivity: 0.7044398
```

```
cat("Standard Deviation of Sensitivity:", sd_sensitivity, "\n")
```

```
## Standard Deviation of Sensitivity: 0.02435222
```

```
cat("Mean Specificity:", mean_specificity, "\n")
```

```
## Mean Specificity: 0.9951951
```

```
cat("Standard Deviation of Specificity:", sd_specificity, "\n")
```

```
## Standard Deviation of Specificity: 0.006203281
```

```
cat("Mean PPV:", mean_ppv, "\n")
```

```
## Mean PPV: 0.9934099
```

```
cat("Standard Deviation of PPV:", sd_ppv, "\n")
```

```
## Standard Deviation of PPV: 0.008514105
```

```
cat("Mean NPV:", mean_npv, "\n")
```

```
## Mean NPV: 0.7687485
```

```
cat("Standard Deviation of NPV:", sd_npv, "\n")
```

```
## Standard Deviation of NPV: 0.01422905
```

```
cat("Mean Accuracy:", mean_accuracy, "\n")
```

```
## Mean Accuracy: 0.8487922
```

```
cat("Standard Deviation of Accuracy:", sd_accuracy, "\n")
```

```
## Standard Deviation of Accuracy: 0.01219834
```

```
# Save the models, confusion matrices, and results table
save(models, file = "models.RData")
save(conf_matrices, file = "conf_matrices.RData")
save(results_table, file = "results_table.RData")
```

# **Testing Classifier 7- RF**

```
library(caret)
library(randomForest)
library(pROC)
library(ROCR)

# Load the model from the file
load("models.RData")

# Select the best model (for example, the seventh model)
best_model <- models[[10]]

# Evaluate the model on a new test set
new_test_data <- data.test
new_test_pred <- predict(best_model, new_test_data, type = "response")

# Extract the predicted probabilities for the default class (assuming "1" is the default class)
new_test_predicted_prob <- new_test_pred$predicted[, "1"]

# Convert the predictions to factors
new_test_predicted_prob2 <- ifelse(new_test_predicted_prob > 0.9, 1, 0)
new_test_predicted_prob2 <- factor(new_test_predicted_prob2, levels = c(1, 0))
new_test_actual <- factor(new_test_data$perdida_bin, levels = c(1, 0))

# Calculate the confusion matrix for the new test set
new_test_confusion <- confusionMatrix(new_test_predicted_prob2, new_test_actual)
print(new_test_confusion)
```

```
## Confusion Matrix and Statistics
## 
##           Reference
## Prediction     1     0
##          1   145    93
##          0    72 23203
##                                          
##                Accuracy : 0.993          
##                  95% CI : (0.9918, 0.994)
##     No Information Rate : 0.9908         
##     P-Value [Acc > NIR] : 0.0001291      
##                                          
##                   Kappa : 0.6338         
##                                          
##  Mcnemar's Test P-Value : 0.1194710      
##                                          
##             Sensitivity : 0.668203       
##             Specificity : 0.996008       
##          Pos Pred Value : 0.609244       
##          Neg Pred Value : 0.996907       
##              Prevalence : 0.009229       
##          Detection Rate : 0.006167       
##    Detection Prevalence : 0.010122       
##       Balanced Accuracy : 0.832105       
##                                          
##        'Positive' Class : 1              
##
```

```
# Calculate the AUC
new_auc_value <- auc(new_test_actual, new_test_predicted_prob)
cat("AUC:", new_auc_value, "\n")
```

```
## AUC: 0.9820621
```

# **Confusion matrix Classifier 7**

```
#Confusion matrix 
draw_confusion_matrix(new_test_confusion)
```

# **Classifier 8-RF. Training and validation**

```
library(caret)
library(randomForestSRC)
library(data.table)  # To create the comparison table

# Perform 10-fold cross-validation on the balanced training set
set.seed(69)
folds <- createFolds(data.train.balanced$perdida_bin, k = 10)
accuracies <- numeric(10)
f1_scores <- numeric(10)
sensitivities <- numeric(10)
specificities <- numeric(10)
ppvs <- numeric(10)  # Positive Predictive Values
npvs <- numeric(10)  # Negative Predictive Values
models <- list()  # List to store the models of each fold
conf_matrices <- list()  # List to store the confusion matrices of each fold

for (i in 1:10) {
    # Segment the data by fold using the which() function
    validationIndexes <- folds[[i]]
    validationData <- data.train.balanced[validationIndexes, ]
    trainData <- data.train.balanced[-validationIndexes, ]

    # Train the Random Forest model
    set.seed(69)
    rf_model <- rfsrc(perdida_bin ~ nefrectomia + perdida + hemodialisi + unidad + terapia + dialisi, 
                             data = trainData, 
                             ntree = 500,          # Number of trees
                             mtry = 2,             # Number of variables to consider at each split
                             nodesize = 20,        # Minimum size of terminal nodes
                             importance = TRUE,    # Calculate variable importance
                             sampsize = 0.3 * nrow(trainData))  # Sample size for each tree
    
    # Store the model
    models[[i]] <- rf_model
    
    # Predict on the validation data
    predicted <- predict(rf_model, validationData, type = "response")
    
    # Extract the predicted probabilities for the default class (assuming "1" is the default class)
    predicted_prob <- predicted$predicted[, "1"]
    
    # Convert probabilities to binary predictions using a threshold of 0.5
    predicted_prob2 <- ifelse(predicted_prob > 0.9, 1, 0)
    predicted_prob2 <- factor(predicted_prob2, levels = c(1, 0))
    validation.actual <- factor(validationData$perdida_bin, levels = c(1, 0))
    
    # Calculate the confusion matrix and extract metrics
    conf_matrix <- confusionMatrix(predicted_prob2, validation.actual)
    conf_matrices[[i]] <- conf_matrix  # Store the confusion matrix
    accuracies[i] <- conf_matrix$overall['Accuracy']
    f1_scores[i] <- conf_matrix$byClass['F1']
    sensitivities[i] <- conf_matrix$byClass['Sensitivity']
    specificities[i] <- conf_matrix$byClass['Specificity']
    ppvs[i] <- conf_matrix$byClass['Pos Pred Value']
    npvs[i] <- conf_matrix$byClass['Neg Pred Value']
}

# Create a data table with the results
results_table <- data.table(
  Fold = 1:10,
  Accuracy = accuracies,
  F1_Score = f1_scores,
  Sensitivity = sensitivities,
  Specificity = specificities,
  PPV = ppvs,
  NPV = npvs
)

# Print the results table
print(results_table)
```

```
##      Fold  Accuracy  F1_Score Sensitivity Specificity       PPV       NPV
##     <int>     <num>     <num>       <num>       <num>     <num>     <num>
##  1:     1 0.8461538 0.8219178   0.7058824   0.9880952 0.9836066 0.7685185
##  2:     2 0.8639053 0.8435374   0.7294118   1.0000000 1.0000000 0.7850467
##  3:     3 0.8502994 0.8251748   0.7023810   1.0000000 1.0000000 0.7685185
##  4:     4 0.8323353 0.8028169   0.6785714   0.9879518 0.9827586 0.7522936
##  5:     5 0.8333333 0.8000000   0.6666667   1.0000000 1.0000000 0.7500000
##  6:     6 0.8682635 0.8513514   0.7500000   0.9879518 0.9843750 0.7961165
##  7:     7 0.8392857 0.8111888   0.6823529   1.0000000 1.0000000 0.7545455
##  8:     8 0.8698225 0.8513514   0.7411765   1.0000000 1.0000000 0.7924528
##  9:     9 0.8452381 0.8219178   0.7058824   0.9879518 0.9836066 0.7663551
## 10:    10 0.8750000 0.8590604   0.7529412   1.0000000 1.0000000 0.7980769
```

```
# Calculate the mean and standard deviation of the metrics
mean_f1 <- mean(f1_scores)
sd_f1 <- sd(f1_scores)
mean_sensitivity <- mean(sensitivities)
sd_sensitivity <- sd(sensitivities)
mean_specificity <- mean(specificities)
sd_specificity <- sd(specificities)
mean_ppv <- mean(ppvs)
sd_ppv <- sd(ppvs)
mean_npv <- mean(npvs)
sd_npv <- sd(npvs)
mean_accuracy <- mean(accuracies)
sd_accuracy <- sd(accuracies)

# Print the summarized results
cat("Mean F1 Score:", mean_f1, "\n")
```

```
## Mean F1 Score: 0.8288317
```

```
cat("Standard Deviation of F1 Score:", sd_f1, "\n")
```

```
## Standard Deviation of F1 Score: 0.02127227
```

```
cat("Mean Sensitivity:", mean_sensitivity, "\n")
```

```
## Mean Sensitivity: 0.7115266
```

```
cat("Standard Deviation of Sensitivity:", sd_sensitivity, "\n")
```

```
## Standard Deviation of Sensitivity: 0.03071605
```

```
cat("Mean Specificity:", mean_specificity, "\n")
```

```
## Mean Specificity: 0.9951951
```

```
cat("Standard Deviation of Specificity:", sd_specificity, "\n")
```

```
## Standard Deviation of Specificity: 0.006203281
```

```
cat("Mean PPV:", mean_ppv, "\n")
```

```
## Mean PPV: 0.9934347
```

```
cat("Standard Deviation of PPV:", sd_ppv, "\n")
```

```
## Standard Deviation of PPV: 0.008484369
```

```
cat("Mean NPV:", mean_npv, "\n")
```

```
## Mean NPV: 0.7731924
```

```
cat("Standard Deviation of NPV:", sd_npv, "\n")
```

```
## Standard Deviation of NPV: 0.0184663
```

```
cat("Mean Accuracy:", mean_accuracy, "\n")
```

```
## Mean Accuracy: 0.8523637
```

```
cat("Standard Deviation of Accuracy:", sd_accuracy, "\n")
```

```
## Standard Deviation of Accuracy: 0.015745
```

```
# Save the models, confusion matrices, and results table
save(models, file = "models.RData")
save(conf_matrices, file = "conf_matrices.RData")
save(results_table, file = "results_table.RData")
```

# **Testing Classifier 8- RF**

```
library(caret)
library(randomForest)
library(pROC)
library(ROCR)

# Load the model from the file
load("models.RData")

# Select the best model (for example, the seventh model)
best_model <- models[[10]]

# Evaluate the model on a new test set
new_test_data <- data.test
new_test_pred <- predict(best_model, new_test_data, type = "response")

# Extract the predicted probabilities for the default class (assuming "1" is the default class)
new_test_predicted_prob <- new_test_pred$predicted[, "1"]

# Convert the predictions to factors
new_test_predicted_prob2 <- ifelse(new_test_predicted_prob > 0.9, 1, 0)
new_test_predicted_prob2 <- factor(new_test_predicted_prob2, levels = c(1, 0))
new_test_actual <- factor(new_test_data$perdida_bin, levels = c(1, 0))

# Calculate the confusion matrix for the new test set
new_test_confusion <- confusionMatrix(new_test_predicted_prob2, new_test_actual)
print(new_test_confusion)
```

```
## Confusion Matrix and Statistics
## 
##           Reference
## Prediction     1     0
##          1   150   105
##          0    67 23191
##                                           
##                Accuracy : 0.9927          
##                  95% CI : (0.9915, 0.9937)
##     No Information Rate : 0.9908          
##     P-Value [Acc > NIR] : 0.0008563       
##                                           
##                   Kappa : 0.6319          
##                                           
##  Mcnemar's Test P-Value : 0.0047841       
##                                           
##             Sensitivity : 0.691244        
##             Specificity : 0.995493        
##          Pos Pred Value : 0.588235        
##          Neg Pred Value : 0.997119        
##              Prevalence : 0.009229        
##          Detection Rate : 0.006379        
##    Detection Prevalence : 0.010845        
##       Balanced Accuracy : 0.843369        
##                                           
##        'Positive' Class : 1               
##
```

```
# Calculate the AUC
new_auc_value <- auc(new_test_actual, new_test_predicted_prob)
cat("AUC:", new_auc_value, "\n")
```

```
## AUC: 0.9823325
```

# **Confusion matrix Classifier 8**

```
#Confusion matrix 
draw_confusion_matrix(new_test_confusion)
```

# **Classifier 9-RF. Training and validation**

```
library(caret)
library(randomForestSRC)
library(data.table)  # To create the comparison table
library(pROC)  # For AUC calculation

# Perform 10-fold cross-validation on the balanced training set
set.seed(69)
folds <- createFolds(data.train.balanced$perdida_bin, k = 10)
accuracies <- numeric(10)
f1_scores <- numeric(10)
sensitivities <- numeric(10)
specificities <- numeric(10)
ppvs <- numeric(10)  # Positive Predictive Values
npvs <- numeric(10)  # Negative Predictive Values
aucs <- numeric(10)  # AUC values
models <- list()  # List to store the models of each fold
conf_matrices <- list()  # List to store the confusion matrices of each fold

for (i in 1:10) {
    # Segment the data by fold using the which() function
    validationIndexes <- folds[[i]]
    validationData <- data.train.balanced[validationIndexes, ]
    trainData <- data.train.balanced[-validationIndexes, ]

    # Train the Random Forest model
    set.seed(69)
    rf_model <- rfsrc(perdida_bin ~ nefrectomia + perdida + hemodialisi + unidad + terapia + dialisi, 
                             data = trainData, 
                             ntree = 500,          # Number of trees
                             mtry = 2,             # Number of variables to consider at each split
                             nodesize = 2,        # Minimum size of terminal nodes
                             nodedepth = 5,        # Maximum depth of the trees
                             importance = TRUE,    # Calculate variable importance
                             sampsize = 0.1 * nrow(trainData))  # Sample size for each tree
    
    # Store the model
    models[[i]] <- rf_model
    
    # Predict on the validation data
    predicted <- predict(rf_model, validationData, type = "response")
    
    # Extract the predicted probabilities for the default class (assuming "1" is the default class)
    predicted_prob <- predicted$predicted[, "1"]
    
    # Convert probabilities to binary predictions using a threshold of 0.5
    predicted_prob2 <- ifelse(predicted_prob > 0.9, 1, 0)
    predicted_prob2 <- factor(predicted_prob2, levels = c(1, 0))
    validation.actual <- factor(validationData$perdida_bin, levels = c(1, 0))
    
    # Calculate the confusion matrix and extract metrics
    conf_matrix <- confusionMatrix(predicted_prob2, validation.actual)
    conf_matrices[[i]] <- conf_matrix  # Store the confusion matrix
    accuracies[i] <- conf_matrix$overall['Accuracy']
    f1_scores[i] <- conf_matrix$byClass['F1']
    sensitivities[i] <- conf_matrix$byClass['Sensitivity']
    specificities[i] <- conf_matrix$byClass['Specificity']
    ppvs[i] <- conf_matrix$byClass['Pos Pred Value']
    npvs[i] <- conf_matrix$byClass['Neg Pred Value']
    
    # Calculate AUC
    roc_obj <- roc(validation.actual, as.numeric(predicted_prob))
    aucs[i] <- auc(roc_obj)
}

# Create a data table with the results
results_table <- data.table(
  Fold = 1:10,
  Accuracy = accuracies,
  F1_Score = f1_scores,
  Sensitivity = sensitivities,
  Specificity = specificities,
  PPV = ppvs,
  NPV = npvs,
  AUC = aucs
)

# Print the results table
print(results_table)
```

```
##      Fold  Accuracy  F1_Score Sensitivity Specificity       PPV       NPV
##     <int>     <num>     <num>       <num>       <num>     <num>     <num>
##  1:     1 0.8461538 0.8219178   0.7058824   0.9880952 0.9836066 0.7685185
##  2:     2 0.8875740 0.8741722   0.7764706   1.0000000 1.0000000 0.8155340
##  3:     3 0.8502994 0.8251748   0.7023810   1.0000000 1.0000000 0.7685185
##  4:     4 0.8502994 0.8275862   0.7142857   0.9879518 0.9836066 0.7735849
##  5:     5 0.8392857 0.8085106   0.6785714   1.0000000 1.0000000 0.7567568
##  6:     6 0.8802395 0.8666667   0.7738095   0.9879518 0.9848485 0.8118812
##  7:     7 0.8630952 0.8435374   0.7294118   1.0000000 1.0000000 0.7830189
##  8:     8 0.8698225 0.8513514   0.7411765   1.0000000 1.0000000 0.7924528
##  9:     9 0.8690476 0.8533333   0.7529412   0.9879518 0.9846154 0.7961165
## 10:    10 0.8809524 0.8666667   0.7647059   1.0000000 1.0000000 0.8058252
##           AUC
##         <num>
##  1: 0.9700280
##  2: 0.9892857
##  3: 0.9955536
##  4: 0.9735370
##  5: 0.9829223
##  6: 0.9676563
##  7: 0.9847626
##  8: 0.9940476
##  9: 0.9878809
## 10: 0.9866052
```

```
# Calculate the mean and standard deviation of the metrics
mean_f1 <- mean(f1_scores)
sd_f1 <- sd(f1_scores)
mean_sensitivity <- mean(sensitivities)
sd_sensitivity <- sd(sensitivities)
mean_specificity <- mean(specificities)
sd_specificity <- sd(specificities)
mean_ppv <- mean(ppvs)
sd_ppv <- sd(ppvs)
mean_npv <- mean(npvs)
sd_npv <- sd(npvs)
mean_accuracy <- mean(accuracies)
sd_accuracy <- sd(accuracies)
mean_auc <- mean(aucs)
sd_auc <- sd(aucs)

# Print the summarized results
cat("Mean F1 Score:", mean_f1, "\n")
```

```
## Mean F1 Score: 0.8438917
```

```
cat("Standard Deviation of F1 Score:", sd_f1, "\n")
```

```
## Standard Deviation of F1 Score: 0.02223632
```

```
cat("Mean Sensitivity:", mean_sensitivity, "\n")
```

```
## Mean Sensitivity: 0.7339636
```

```
cat("Standard Deviation of Sensitivity:", sd_sensitivity, "\n")
```

```
## Standard Deviation of Sensitivity: 0.03335675
```

```
cat("Mean Specificity:", mean_specificity, "\n")
```

```
## Mean Specificity: 0.9951951
```

```
cat("Standard Deviation of Specificity:", sd_specificity, "\n")
```

```
## Standard Deviation of Specificity: 0.006203281
```

```
cat("Mean PPV:", mean_ppv, "\n")
```

```
## Mean PPV: 0.9936677
```

```
cat("Standard Deviation of PPV:", sd_ppv, "\n")
```

```
## Standard Deviation of PPV: 0.008183753
```

```
cat("Mean NPV:", mean_npv, "\n")
```

```
## Mean NPV: 0.7872207
```

```
cat("Standard Deviation of NPV:", sd_npv, "\n")
```

```
## Standard Deviation of NPV: 0.02025835
```

```
cat("Mean Accuracy:", mean_accuracy, "\n")
```

```
## Mean Accuracy: 0.863677
```

```
cat("Standard Deviation of Accuracy:", sd_accuracy, "\n")
```

```
## Standard Deviation of Accuracy: 0.01656866
```

```
cat("Mean AUC:", mean_auc, "\n")
```

```
## Mean AUC: 0.9832279
```

```
cat("Standard Deviation of AUC:", sd_auc, "\n")
```

```
## Standard Deviation of AUC: 0.009728636
```

```
# Save the models, confusion matrices, and results table
save(models, file = "models.RData")
save(conf_matrices, file = "conf_matrices.RData")
save(results_table, file = "results_table.RData")
```

# **Testing Classifier 9- RF**

```
library(caret)
library(randomForest)
library(pROC)
library(ROCR)

# Load the model from the file
load("models.RData")

# Select the best model (for example, the seventh model)
best_model <- models[[6]]

# Evaluate the model on a new test set
new_test_data <- data.test
new_test_pred <- predict(best_model, new_test_data, type = "response")

# Extract the predicted probabilities for the default class (assuming "1" is the default class)
new_test_predicted_prob <- new_test_pred$predicted[, "1"]

# Convert the predictions to factors
new_test_predicted_prob2 <- ifelse(new_test_predicted_prob > 0.9, 1, 0)
new_test_predicted_prob2 <- factor(new_test_predicted_prob2, levels = c(1, 0))
new_test_actual <- factor(new_test_data$perdida_bin, levels = c(1, 0))

# Calculate the confusion matrix for the new test set
new_test_confusion <- confusionMatrix(new_test_predicted_prob2, new_test_actual)
print(new_test_confusion)
```

```
## Confusion Matrix and Statistics
## 
##           Reference
## Prediction     1     0
##          1   165   125
##          0    52 23171
##                                           
##                Accuracy : 0.9925          
##                  95% CI : (0.9913, 0.9935)
##     No Information Rate : 0.9908          
##     P-Value [Acc > NIR] : 0.002801        
##                                           
##                   Kappa : 0.6472          
##                                           
##  Mcnemar's Test P-Value : 6.238e-08       
##                                           
##             Sensitivity : 0.760369        
##             Specificity : 0.994634        
##          Pos Pred Value : 0.568966        
##          Neg Pred Value : 0.997761        
##              Prevalence : 0.009229        
##          Detection Rate : 0.007017        
##    Detection Prevalence : 0.012334        
##       Balanced Accuracy : 0.877501        
##                                           
##        'Positive' Class : 1               
##
```

```
# Calculate the AUC
new_auc_value <- auc(new_test_actual, new_test_predicted_prob)
```

```
## Setting levels: control = 1, case = 0
```

```
## Setting direction: controls > cases
```

```
cat("AUC:", new_auc_value, "\n")
```

```
## AUC: 0.9832154
```

# **Confusion matrix Classifier 9**

```
#Confusion matrix 
draw_confusion_matrix(new_test_confusion)
```

```
#importancia de las variables
print(vimp(best_model)$importance)
```

```
##                   all           1         0
## nefrectomia 0.6285129  0.84823235 2.5813181
## perdida     0.4642036  0.06677984 2.4743966
## hemodialisi 0.4623397  0.02061899 2.5110264
## unidad      0.2472307  0.01112997 1.3426358
## terapia     0.1087773 -0.02275937 0.6187982
## dialisi     0.2918572  0.02432898 1.5736354
```

```
#plot de importancia de las variables 

# Cargar la biblioteca ggplot2
library(ggplot2)

# Cargar la biblioteca ggplot2
library(ggplot2)

# Crear un data frame con los datos
datos <- data.frame(
  terminos = c("Nefrectomia", "Perdida", "Hemodialisis", "Unidad", "Terapia", "Dialisis"),
  values = c(0.6285129, 0.4642036, 0.4623397, 0.2472307, 0.1087773 , 0.2918572)
)

# Convertir los valores a porcentajes
datos$valores <- datos$values * 100

# Ordenar los datos en orden descendente
datos <- datos[order(-datos$values),]

# Crear el gráfico de barras con degradado azul
p<-ggplot(datos, aes(x = reorder(terminos, values), y = valores, fill = values)) +
  geom_bar(stat = "identity") +
  coord_flip() +
  scale_fill_gradient(low = "lightblue", high = "blue") +
  theme_minimal() +
  theme(plot.title = element_text(face = "bold")) +
  labs(title = "VARIABLE IMPORTANCE", cex.main=2,
       x = "",
       y = "Relative importance(%)")
p
```

# **Neural networks**

## **Data Partition- training, validation and test sets**

```
# Install and load the necessary libraries
#install.packages("ROSE")
#install.packages("caret")
library(ROSE)
library(caret)
library(glmnet)

# Check the distribution of the target variable
table(data.lasso$perdida_bin)
```

```
## 
##      0      1 
## 116503   1063
```

```
# Split the data into training (80%) and test (20%) sets
set.seed(69)
trainIndex <- createDataPartition(data.lasso$perdida_bin, p = 0.8, list = FALSE)
data.train <- data.lasso[trainIndex, ]
data.test <- data.lasso[-trainIndex, ]

# Check the distribution of the target variable in the test set
table(data.test$perdida_bin)
```

```
## 
##     0     1 
## 23296   217
```

```
# Apply undersampling to the training set to balance it 
data.train.balanced <- ovun.sample(perdida_bin ~ ., data = data.train, method = "under", seed = 69)$data

# Check the new distribution of the target variable in the balanced training set
table(data.train.balanced$perdida_bin)
```

```
## 
##   0   1 
## 834 846
```

```
# Libraries
library(tidyverse)
#library(keras)
library(mlbench)
library(dplyr)
library(magrittr)
library(neuralnet)
#library(tensorflow)
```

# **Classifier 10- NN. Training and validation**

```
library(caret)
library(neuralnet)
library(data.table)  # Para crear la tabla comparativa

# Perform 10-fold cross-validation on the balanced training set
set.seed(69)
folds <- createFolds(data.train.balanced$perdida_bin, k = 10)
accuracies <- numeric(10)
f1_scores <- numeric(10)
sensitivities <- numeric(10)
specificities <- numeric(10)
ppvs <- numeric(10)  # Positive Predictive Values
npvs <- numeric(10)  # Negative Predictive Values
models <- list()  # Lista para almacenar los modelos de cada fold
conf_matrices <- list()  # Lista para almacenar las matrices de confusión de cada fold

for (i in 1:10) {
    # Segment your data by fold using the which() function 
    validationIndexes <- folds[[i]]
    validationData <- data.train.balanced[validationIndexes, ]
    trainData <- data.train.balanced[-validationIndexes, ]

    # Train the neural network model
    set.seed(69)
    n <- neuralnet(perdida_bin ~ nefrectomia + perdida + hemodialisi + unidad + terapia + dialisi,
                   data = trainData,
                   hidden = c(4, 2),
                   linear.output = FALSE,
                   lifesign = 'full',
                   threshold = 0.07,
                   lifesign.step = 10,
                   rep = 1) 
    
    # Store the model
    models[[i]] <- n
    
    # Predict on the validation data
    predicted <- predict(n, validationData, type = "response")
 
    # Convert probabilities to binary predictions using a threshold of 0.1
    predicted_prob2 <- ifelse(predicted[, 1] > 0.1, 1, 0)
    predicted_prob2 <- factor(predicted_prob2, levels = c(1, 0))
    validation.actual <- factor(validationData$perdida_bin, levels = c(1, 0))
    
    # Compute the confusion matrix and extract metrics
    conf_matrix <- confusionMatrix(predicted_prob2, validation.actual)
    conf_matrices[[i]] <- conf_matrix  # Store the confusion matrix
    accuracies[i] <- conf_matrix$overall['Accuracy']
    f1_scores[i] <- conf_matrix$byClass['F1']
    sensitivities[i] <- conf_matrix$byClass['Sensitivity']
    specificities[i] <- conf_matrix$byClass['Specificity']
    ppvs[i] <- conf_matrix$byClass['Pos Pred Value']
    npvs[i] <- conf_matrix$byClass['Neg Pred Value']
}

# Create a data table with the results
results_table <- data.table(
  Fold = 1:10,
  Accuracy = accuracies,
  F1_Score = f1_scores,
  Sensitivity = sensitivities,
  Specificity = specificities,
  PPV = ppvs,
  NPV = npvs
)

# Print the results table
print(results_table)
```

```
##      Fold  Accuracy  F1_Score Sensitivity Specificity       PPV       NPV
##     <int>     <num>     <num>       <num>       <num>     <num>     <num>
##  1:     1 0.9285714 0.9325843   0.9651163   0.8902439 0.9021739 0.9605263
##  2:     2 0.9523810 0.9550562   1.0000000   0.9036145 0.9139785 1.0000000
##  3:     3 0.9404762 0.9456522   0.9560440   0.9220779 0.9354839 0.9466667
##  4:     4 0.9583333 0.9651741   0.9897959   0.9142857 0.9417476 0.9846154
##  5:     5 0.9702381 0.9677419   0.9868421   0.9565217 0.9493671 0.9887640
##  6:     6 0.9464286 0.9433962   0.9868421   0.9130435 0.9036145 0.9882353
##  7:     7 0.9285714 0.9250000   1.0000000   0.8723404 0.8604651 1.0000000
##  8:     8 0.9464286 0.9491525   0.9882353   0.9036145 0.9130435 0.9868421
##  9:     9 0.9642857 0.9662921   0.9662921   0.9620253 0.9662921 0.9620253
## 10:    10 0.9583333 0.9604520   0.9883721   0.9268293 0.9340659 0.9870130
```

```
# Calculate the mean and standard deviation of the metrics
mean_f1 <- mean(f1_scores)
sd_f1 <- sd(f1_scores)
mean_sensitivity <- mean(sensitivities)
sd_sensitivity <- sd(sensitivities)
mean_specificity <- mean(specificities)
sd_specificity <- sd(specificities)
mean_ppv <- mean(ppvs)
sd_ppv <- sd(ppvs)
mean_npv <- mean(npvs)
sd_npv <- sd(npvs)
mean_accuracy <- mean(accuracies)
sd_accuracy <- sd(accuracies)

# Print the summary results
cat("Mean F1 Score:", mean_f1, "\n")
```

```
## Mean F1 Score: 0.9510502
```

```
cat("Standard Deviation of F1 Score:", sd_f1, "\n")
```

```
## Standard Deviation of F1 Score: 0.01462792
```

```
cat("Mean Sensitivity:", mean_sensitivity, "\n")
```

```
## Mean Sensitivity: 0.982754
```

```
cat("Standard Deviation of Sensitivity:", sd_sensitivity, "\n")
```

```
## Standard Deviation of Sensitivity: 0.01503691
```

```
cat("Mean Specificity:", mean_specificity, "\n")
```

```
## Mean Specificity: 0.9164597
```

```
cat("Standard Deviation of Specificity:", sd_specificity, "\n")
```

```
## Standard Deviation of Specificity: 0.0274883
```

```
cat("Mean PPV:", mean_ppv, "\n")
```

```
## Mean PPV: 0.9220232
```

```
cat("Standard Deviation of PPV:", sd_ppv, "\n")
```

```
## Standard Deviation of PPV: 0.02998207
```

```
cat("Mean NPV:", mean_npv, "\n")
```

```
## Mean NPV: 0.9804688
```

```
cat("Standard Deviation of NPV:", sd_npv, "\n")
```

```
## Standard Deviation of NPV: 0.01786727
```

```
cat("Mean Accuracy:", mean_accuracy, "\n")
```

```
## Mean Accuracy: 0.9494048
```

```
cat("Standard Deviation of Accuracy:", sd_accuracy, "\n")
```

```
## Standard Deviation of Accuracy: 0.01409987
```

```
# Save the models, confusion matrices, and results table
save(models, file = "models.RData")
save(conf_matrices, file = "conf_matrices.RData")
save(results_table, file = "results_table.RData")
```

# **Testing Classifier 10-NN**

```
library(caret)
library(neuralnet)
library(pROC)
library(ROCR)

# Cargar el modelo desde el archivo
load("models.RData")

# Seleccionar el mejor modelo (por ejemplo, el primer modelo)
best_model <- models[[7]]

# Evaluar el modelo en un nuevo conjunto de prueba
new_test_data <- data.test
new_test_pred <- predict(best_model, new_test_data, type = "response")

# Extraer las probabilidades predichas
new_test_predicted_prob <- new_test_pred[, 1]
new_test_predicted_prob <- as.numeric(new_test_predicted_prob)

# Convertir probabilidades a predicciones binarias usando un umbral de 0.9
new_test_predicted_prob2 <- ifelse(new_test_predicted_prob > 0.9, 1, 0)
new_test_predicted_prob2 <- factor(new_test_predicted_prob2, levels = c(1, 0))
new_test_actual <- factor(new_test_data$perdida_bin, levels = c(1, 0))

# Calcular la matriz de confusión para el nuevo conjunto de prueba
new_test_confusion <- confusionMatrix(new_test_predicted_prob2, new_test_actual)
print(new_test_confusion)
```

```
## Confusion Matrix and Statistics
## 
##           Reference
## Prediction     1     0
##          1   179   237
##          0    38 23059
##                                           
##                Accuracy : 0.9883          
##                  95% CI : (0.9868, 0.9896)
##     No Information Rate : 0.9908          
##     P-Value [Acc > NIR] : 0.9999          
##                                           
##                   Kappa : 0.5602          
##                                           
##  Mcnemar's Test P-Value : <2e-16          
##                                           
##             Sensitivity : 0.824885        
##             Specificity : 0.989827        
##          Pos Pred Value : 0.430288        
##          Neg Pred Value : 0.998355        
##              Prevalence : 0.009229        
##          Detection Rate : 0.007613        
##    Detection Prevalence : 0.017692        
##       Balanced Accuracy : 0.907356        
##                                           
##        'Positive' Class : 1               
##
```

```
# Calcular el AUC
new_auc_value <- auc(new_test_actual, new_test_predicted_prob)
cat("AUC:", new_auc_value, "\n")
```

```
## AUC: 0.9826893
```

# **Confusion matrix Classifier 10**

```
#Confusion matrix 
draw_confusion_matrix(new_test_confusion)
```

# **Classifier 11- NN. Training and validation**

```
library(caret)
library(neuralnet)
library(data.table)  # Para crear la tabla comparativa

# Perform 10-fold cross-validation on the balanced training set
set.seed(69)
folds <- createFolds(data.train.balanced$perdida_bin, k = 10)
accuracies <- numeric(10)
f1_scores <- numeric(10)
sensitivities <- numeric(10)
specificities <- numeric(10)
ppvs <- numeric(10)  # Positive Predictive Values
npvs <- numeric(10)  # Negative Predictive Values
models <- list()  # Lista para almacenar los modelos de cada fold
conf_matrices <- list()  # Lista para almacenar las matrices de confusión de cada fold

for (i in 1:10) {
    # Segment your data by fold using the which() function 
    validationIndexes <- folds[[i]]
    validationData <- data.train.balanced[validationIndexes, ]
    trainData <- data.train.balanced[-validationIndexes, ]

    # Train the neural network model
    set.seed(69)
    set.seed(69)
    n1 <- neuralnet(perdida_bin ~ nefrectomia+ perdida+ hemodialisi+ unidad+ terapia+ dialisi,
               data = trainData,
               hidden = c(6,2),
               linear.output = F,
               lifesign = 'full',
               threshold = 0.05,
               lifesign.step = 10,
               rep=1)

    
    # Store the model
    models[[i]] <- n1
    
    # Predict on the validation data
    predicted <- predict(n1, validationData, type = "response")
 
    # Convert probabilities to binary predictions using a threshold of 0.1
    predicted_prob2 <- ifelse(predicted[, 1] > 0.9, 1, 0)
    predicted_prob2 <- factor(predicted_prob2, levels = c(1, 0))
    validation.actual <- factor(validationData$perdida_bin, levels = c(1, 0))
    
    # Compute the confusion matrix and extract metrics
    conf_matrix <- confusionMatrix(predicted_prob2, validation.actual)
    conf_matrices[[i]] <- conf_matrix  # Store the confusion matrix
    accuracies[i] <- conf_matrix$overall['Accuracy']
    f1_scores[i] <- conf_matrix$byClass['F1']
    sensitivities[i] <- conf_matrix$byClass['Sensitivity']
    specificities[i] <- conf_matrix$byClass['Specificity']
    ppvs[i] <- conf_matrix$byClass['Pos Pred Value']
    npvs[i] <- conf_matrix$byClass['Neg Pred Value']
}

# Create a data table with the results
results_table <- data.table(
  Fold = 1:10,
  Accuracy = accuracies,
  F1_Score = f1_scores,
  Sensitivity = sensitivities,
  Specificity = specificities,
  PPV = ppvs,
  NPV = npvs
)

# Print the results table
print(results_table)
```

```
##      Fold  Accuracy  F1_Score Sensitivity Specificity       PPV       NPV
##     <int>     <num>     <num>       <num>       <num>     <num>     <num>
##  1:     1 0.9226190 0.9192547   0.8604651   0.9878049 0.9866667 0.8709677
##  2:     2 0.9285714 0.9250000   0.8705882   0.9879518 0.9866667 0.8817204
##  3:     3 0.8928571 0.8941176   0.8351648   0.9610390 0.9620253 0.8314607
##  4:     4 0.9226190 0.9312169   0.8979592   0.9571429 0.9670330 0.8701299
##  5:     5 0.9166667 0.8985507   0.8157895   1.0000000 1.0000000 0.8679245
##  6:     6 0.9345238 0.9230769   0.8684211   0.9891304 0.9850746 0.9009901
##  7:     7 0.9345238 0.9208633   0.8648649   0.9893617 0.9846154 0.9029126
##  8:     8 0.9226190 0.9192547   0.8705882   0.9759036 0.9736842 0.8804348
##  9:     9 0.9047619 0.9012346   0.8202247   1.0000000 1.0000000 0.8315789
## 10:    10 0.9404762 0.9390244   0.8953488   0.9878049 0.9871795 0.9000000
```

```
# Calculate the mean and standard deviation of the metrics
mean_f1 <- mean(f1_scores)
sd_f1 <- sd(f1_scores)
mean_sensitivity <- mean(sensitivities)
sd_sensitivity <- sd(sensitivities)
mean_specificity <- mean(specificities)
sd_specificity <- sd(specificities)
mean_ppv <- mean(ppvs)
sd_ppv <- sd(ppvs)
mean_npv <- mean(npvs)
sd_npv <- sd(npvs)
mean_accuracy <- mean(accuracies)
sd_accuracy <- sd(accuracies)

# Print the summary results
cat("Mean F1 Score:", mean_f1, "\n")
```

```
## Mean F1 Score: 0.9171594
```

```
cat("Standard Deviation of F1 Score:", sd_f1, "\n")
```

```
## Standard Deviation of F1 Score: 0.0146268
```

```
cat("Mean Sensitivity:", mean_sensitivity, "\n")
```

```
## Mean Sensitivity: 0.8599415
```

```
cat("Standard Deviation of Sensitivity:", sd_sensitivity, "\n")
```

```
## Standard Deviation of Sensitivity: 0.02821121
```

```
cat("Mean Specificity:", mean_specificity, "\n")
```

```
## Mean Specificity: 0.9836139
```

```
cat("Standard Deviation of Specificity:", sd_specificity, "\n")
```

```
## Standard Deviation of Specificity: 0.01462196
```

```
cat("Mean PPV:", mean_ppv, "\n")
```

```
## Mean PPV: 0.9832945
```

```
cat("Standard Deviation of PPV:", sd_ppv, "\n")
```

```
## Standard Deviation of PPV: 0.01251697
```

```
cat("Mean NPV:", mean_npv, "\n")
```

```
## Mean NPV: 0.873812
```

```
cat("Standard Deviation of NPV:", sd_npv, "\n")
```

```
## Standard Deviation of NPV: 0.02584416
```

```
cat("Mean Accuracy:", mean_accuracy, "\n")
```

```
## Mean Accuracy: 0.9220238
```

```
cat("Standard Deviation of Accuracy:", sd_accuracy, "\n")
```

```
## Standard Deviation of Accuracy: 0.01443103
```

```
# Save the models, confusion matrices, and results table
save(models, file = "models.RData")
save(conf_matrices, file = "conf_matrices.RData")
save(results_table, file = "results_table.RData")
```

# **Testing Classifier 11- NN**

```
library(caret)
library(neuralnet)
library(pROC)
library(ROCR)

# Cargar el modelo desde el archivo
load("models.RData")

# Seleccionar el mejor modelo (por ejemplo, el primer modelo)
best_model <- models[[9]]

# Evaluar el modelo en un nuevo conjunto de prueba
new_test_data <- data.test
new_test_pred <- predict(best_model, new_test_data, type = "response")

# Extraer las probabilidades predichas
new_test_predicted_prob <- new_test_pred[, 1]
new_test_predicted_prob <- as.numeric(new_test_predicted_prob)

# Convertir probabilidades a predicciones binarias usando un umbral de 0.9
new_test_predicted_prob2 <- ifelse(new_test_predicted_prob > 0.9, 1, 0)
new_test_predicted_prob2 <- factor(new_test_predicted_prob2, levels = c(1, 0))
new_test_actual <- factor(new_test_data$perdida_bin, levels = c(1, 0))

# Calcular la matriz de confusión para el nuevo conjunto de prueba
new_test_confusion <- confusionMatrix(new_test_predicted_prob2, new_test_actual)
print(new_test_confusion)
```

```
## Confusion Matrix and Statistics
## 
##           Reference
## Prediction     1     0
##          1   178   230
##          0    39 23066
##                                           
##                Accuracy : 0.9886          
##                  95% CI : (0.9871, 0.9899)
##     No Information Rate : 0.9908          
##     P-Value [Acc > NIR] : 0.9997          
##                                           
##                   Kappa : 0.5644          
##                                           
##  Mcnemar's Test P-Value : <2e-16          
##                                           
##             Sensitivity : 0.820276        
##             Specificity : 0.990127        
##          Pos Pred Value : 0.436275        
##          Neg Pred Value : 0.998312        
##              Prevalence : 0.009229        
##          Detection Rate : 0.007570        
##    Detection Prevalence : 0.017352        
##       Balanced Accuracy : 0.905202        
##                                           
##        'Positive' Class : 1               
##
```

```
# Calcular el AUC
new_auc_value <- auc(new_test_actual, new_test_predicted_prob)
cat("AUC:", new_auc_value, "\n")
```

```
## AUC: 0.9830185
```

# **Confusion matrix Classifier 11**

```
#Confusion matrix 
draw_confusion_matrix(new_test_confusion)
```

# **Classifier 12- NN. Training and validation**

```
library(caret)
library(neuralnet)
library(data.table)  # Para crear la tabla comparativa

# Perform 10-fold cross-validation on the balanced training set
set.seed(69)
folds <- createFolds(data.train.balanced$perdida_bin, k = 10)
accuracies <- numeric(10)
f1_scores <- numeric(10)
sensitivities <- numeric(10)
specificities <- numeric(10)
ppvs <- numeric(10)  # Positive Predictive Values
npvs <- numeric(10)  # Negative Predictive Values
models <- list()  # Lista para almacenar los modelos de cada fold
conf_matrices <- list()  # Lista para almacenar las matrices de confusión de cada fold

for (i in 1:10) {
    # Segment your data by fold using the which() function 
    validationIndexes <- folds[[i]]
    validationData <- data.train.balanced[validationIndexes, ]
    trainData <- data.train.balanced[-validationIndexes, ]

    # Train the neural network model
    set.seed(69)
    n2 <- neuralnet(perdida_bin ~ nefrectomia+ perdida+ hemodialisi+ unidad+ terapia+ dialisi,
               data = trainData,
               hidden = c(2,2),
               linear.output = F,
               lifesign = 'full',
               threshold = 0.05,
               lifesign.step = 10,
               rep=1)

    
    # Store the model
    models[[i]] <- n2
    
    # Predict on the validation data
    predicted <- predict(n2, validationData, type = "response")
 
    # Convert probabilities to binary predictions using a threshold of 0.1
    predicted_prob2 <- ifelse(predicted[, 1] > 0.9, 1, 0)
    predicted_prob2 <- factor(predicted_prob2, levels = c(1, 0))
    validation.actual <- factor(validationData$perdida_bin, levels = c(1, 0))
    
    # Compute the confusion matrix and extract metrics
    conf_matrix <- confusionMatrix(predicted_prob2, validation.actual)
    conf_matrices[[i]] <- conf_matrix  # Store the confusion matrix
    accuracies[i] <- conf_matrix$overall['Accuracy']
    f1_scores[i] <- conf_matrix$byClass['F1']
    sensitivities[i] <- conf_matrix$byClass['Sensitivity']
    specificities[i] <- conf_matrix$byClass['Specificity']
    ppvs[i] <- conf_matrix$byClass['Pos Pred Value']
    npvs[i] <- conf_matrix$byClass['Neg Pred Value']
}

# Create a data table with the results
results_table <- data.table(
  Fold = 1:10,
  Accuracy = accuracies,
  F1_Score = f1_scores,
  Sensitivity = sensitivities,
  Specificity = specificities,
  PPV = ppvs,
  NPV = npvs
)

# Print the results table
print(results_table)
```

```
##      Fold  Accuracy  F1_Score Sensitivity Specificity       PPV       NPV
##     <int>     <num>     <num>       <num>       <num>     <num>     <num>
##  1:     1 0.9285714 0.9259259   0.8720930   0.9878049 0.9868421 0.8804348
##  2:     2 0.9166667 0.9113924   0.8470588   0.9879518 0.9863014 0.8631579
##  3:     3 0.8988095 0.9005848   0.8461538   0.9610390 0.9625000 0.8409091
##  4:     4 0.9345238 0.9417989   0.9081633   0.9714286 0.9780220 0.8831169
##  5:     5 0.9285714 0.9154930   0.8552632   0.9891304 0.9848485 0.8921569
##  6:     6 0.9523810 0.9452055   0.9078947   0.9891304 0.9857143 0.9285714
##  7:     7 0.9226190 0.9064748   0.8513514   0.9787234 0.9692308 0.8932039
##  8:     8 0.9285714 0.9250000   0.8705882   0.9879518 0.9866667 0.8817204
##  9:     9 0.9107143 0.9090909   0.8426966   0.9873418 0.9868421 0.8478261
## 10:    10 0.9345238 0.9325153   0.8837209   0.9878049 0.9870130 0.8901099
```

```
# Calculate the mean and standard deviation of the metrics
mean_f1 <- mean(f1_scores)
sd_f1 <- sd(f1_scores)
mean_sensitivity <- mean(sensitivities)
sd_sensitivity <- sd(sensitivities)
mean_specificity <- mean(specificities)
sd_specificity <- sd(specificities)
mean_ppv <- mean(ppvs)
sd_ppv <- sd(ppvs)
mean_npv <- mean(npvs)
sd_npv <- sd(npvs)
mean_accuracy <- mean(accuracies)
sd_accuracy <- sd(accuracies)

# Print the summary results
cat("Mean F1 Score:", mean_f1, "\n")
```

```
## Mean F1 Score: 0.9213482
```

```
cat("Standard Deviation of F1 Score:", sd_f1, "\n")
```

```
## Standard Deviation of F1 Score: 0.01521103
```

```
cat("Mean Sensitivity:", mean_sensitivity, "\n")
```

```
## Mean Sensitivity: 0.8684984
```

```
cat("Standard Deviation of Sensitivity:", sd_sensitivity, "\n")
```

```
## Standard Deviation of Sensitivity: 0.0246492
```

```
cat("Mean Specificity:", mean_specificity, "\n")
```

```
## Mean Specificity: 0.9828307
```

```
cat("Standard Deviation of Specificity:", sd_specificity, "\n")
```

```
## Standard Deviation of Specificity: 0.009565086
```

```
cat("Mean PPV:", mean_ppv, "\n")
```

```
## Mean PPV: 0.9813981
```

```
cat("Standard Deviation of PPV:", sd_ppv, "\n")
```

```
## Standard Deviation of PPV: 0.008754149
```

```
cat("Mean NPV:", mean_npv, "\n")
```

```
## Mean NPV: 0.8801207
```

```
cat("Standard Deviation of NPV:", sd_npv, "\n")
```

```
## Standard Deviation of NPV: 0.02504032
```

```
cat("Mean Accuracy:", mean_accuracy, "\n")
```

```
## Mean Accuracy: 0.9255952
```

```
cat("Standard Deviation of Accuracy:", sd_accuracy, "\n")
```

```
## Standard Deviation of Accuracy: 0.01464764
```

```
# Save the models, confusion matrices, and results table
save(models, file = "models.RData")
save(conf_matrices, file = "conf_matrices.RData")
save(results_table, file = "results_table.RData")
```

# **Testing Classifier 12- NN**

```
library(caret)
library(neuralnet)
library(pROC)
library(ROCR)

# Cargar el modelo desde el archivo
load("models.RData")

# Seleccionar el mejor modelo (por ejemplo, el primer modelo)
best_model <- models[[10]]

# Evaluar el modelo en un nuevo conjunto de prueba
new_test_data <- data.test
new_test_pred <- predict(best_model, new_test_data, type = "response")

# Extraer las probabilidades predichas
new_test_predicted_prob <- new_test_pred[, 1]
new_test_predicted_prob <- as.numeric(new_test_predicted_prob)

# Convertir probabilidades a predicciones binarias usando un umbral de 0.9
new_test_predicted_prob2 <- ifelse(new_test_predicted_prob > 0.9, 1, 0)
new_test_predicted_prob2 <- factor(new_test_predicted_prob2, levels = c(1, 0))
new_test_actual <- factor(new_test_data$perdida_bin, levels = c(1, 0))

# Calcular la matriz de confusión para el nuevo conjunto de prueba
new_test_confusion <- confusionMatrix(new_test_predicted_prob2, new_test_actual)
print(new_test_confusion)
```

```
## Confusion Matrix and Statistics
## 
##           Reference
## Prediction     1     0
##          1   172   358
##          0    45 22938
##                                           
##                Accuracy : 0.9829          
##                  95% CI : (0.9811, 0.9845)
##     No Information Rate : 0.9908          
##     P-Value [Acc > NIR] : 1               
##                                           
##                   Kappa : 0.4533          
##                                           
##  Mcnemar's Test P-Value : <2e-16          
##                                           
##             Sensitivity : 0.792627        
##             Specificity : 0.984633        
##          Pos Pred Value : 0.324528        
##          Neg Pred Value : 0.998042        
##              Prevalence : 0.009229        
##          Detection Rate : 0.007315        
##    Detection Prevalence : 0.022541        
##       Balanced Accuracy : 0.888630        
##                                           
##        'Positive' Class : 1               
##
```

```
# Calcular el AUC
new_auc_value <- auc(new_test_actual, new_test_predicted_prob)
cat("AUC:", new_auc_value, "\n")
```

```
## AUC: 0.9820716
```

# **Confusion matrix Classifier 12**

```
#Confusion matrix 
draw_confusion_matrix(new_test_confusion)
```

# **Classifier 13- NN. Training and validation**

```
library(caret)
library(neuralnet)
library(data.table)  # Para crear la tabla comparativa
library(pROC)  # Para el cálculo del AUC

# Perform 10-fold cross-validation on the balanced training set
set.seed(69)
folds <- createFolds(data.train.balanced$perdida_bin, k = 10)
accuracies <- numeric(10)
f1_scores <- numeric(10)
sensitivities <- numeric(10)
specificities <- numeric(10)
ppvs <- numeric(10)  # Positive Predictive Values
npvs <- numeric(10)  # Negative Predictive Values
aucs <- numeric(10)  # AUC values
models <- list()  # Lista para almacenar los modelos de cada fold
conf_matrices <- list()  # Lista para almacenar las matrices de confusión de cada fold

for (i in 1:10) {
    # Segment your data by fold using the which() function 
    validationIndexes <- folds[[i]]
    validationData <- data.train.balanced[validationIndexes, ]
    trainData <- data.train.balanced[-validationIndexes, ]

    # Train the neural network model
    set.seed(69)
    n4 <- neuralnet(perdida_bin ~ nefrectomia+ perdida+ hemodialisi+ unidad+ terapia+ dialisi,
               data = trainData,
               hidden = c(4,2),
               linear.output = F,
               lifesign = 'full',
               threshold = 0.03,
               lifesign.step = 10,
               rep=1)

    # Store the model
    models[[i]] <- n4
    
    # Predict on the validation data
    predicted <- predict(n4, validationData, type = "response")
 
    # Convert probabilities to binary predictions using a threshold of 0.1
    predicted_prob2 <- ifelse(predicted[, 1] > 0.9, 1, 0)
    predicted_prob2 <- factor(predicted_prob2, levels = c(1, 0))
    validation.actual <- factor(validationData$perdida_bin, levels = c(1, 0))
    
    # Compute the confusion matrix and extract metrics
    conf_matrix <- confusionMatrix(predicted_prob2, validation.actual)
    conf_matrices[[i]] <- conf_matrix  # Store the confusion matrix
    accuracies[i] <- conf_matrix$overall['Accuracy']
    f1_scores[i] <- conf_matrix$byClass['F1']
    sensitivities[i] <- conf_matrix$byClass['Sensitivity']
    specificities[i] <- conf_matrix$byClass['Specificity']
    ppvs[i] <- conf_matrix$byClass['Pos Pred Value']
    npvs[i] <- conf_matrix$byClass['Neg Pred Value']
    
    # Calculate AUC
    roc_obj <- roc(validation.actual, as.numeric(predicted[, 1]))
    aucs[i] <- auc(roc_obj)
}

# Create a data table with the results
results_table <- data.table(
  Fold = 1:10,
  Accuracy = accuracies,
  F1_Score = f1_scores,
  Sensitivity = sensitivities,
  Specificity = specificities,
  PPV = ppvs,
  NPV = npvs,
  AUC = aucs
)

# Print the results table
print(results_table)
```

```
##      Fold  Accuracy  F1_Score Sensitivity Specificity       PPV       NPV
##     <int>     <num>     <num>       <num>       <num>     <num>     <num>
##  1:     1 0.9285714 0.9259259   0.8720930   0.9878049 0.9868421 0.8804348
##  2:     2 0.9226190 0.9182390   0.8588235   0.9879518 0.9864865 0.8723404
##  3:     3 0.8928571 0.8928571   0.8241758   0.9740260 0.9740260 0.8241758
##  4:     4 0.9166667 0.9255319   0.8877551   0.9571429 0.9666667 0.8589744
##  5:     5 0.9047619 0.8823529   0.7894737   1.0000000 1.0000000 0.8518519
##  6:     6 0.9285714 0.9154930   0.8552632   0.9891304 0.9848485 0.8921569
##  7:     7 0.9226190 0.9051095   0.8378378   0.9893617 0.9841270 0.8857143
##  8:     8 0.9166667 0.9113924   0.8470588   0.9879518 0.9863014 0.8631579
##  9:     9 0.9047619 0.9012346   0.8202247   1.0000000 1.0000000 0.8315789
## 10:    10 0.9404762 0.9390244   0.8953488   0.9878049 0.9871795 0.9000000
##           AUC
##         <num>
##  1: 0.9742626
##  2: 0.9924876
##  3: 0.9648923
##  4: 0.9806122
##  5: 0.9925629
##  6: 0.9843392
##  7: 0.9885710
##  8: 0.9864635
##  9: 0.9799460
## 10: 0.9825581
```

```
# Calculate the mean and standard deviation of the metrics
mean_f1 <- mean(f1_scores)
sd_f1 <- sd(f1_scores)
mean_sensitivity <- mean(sensitivities)
sd_sensitivity <- sd(sensitivities)
mean_specificity <- mean(specificities)
sd_specificity <- sd(specificities)
mean_ppv <- mean(ppvs)
sd_ppv <- sd(ppvs)
mean_npv <- mean(npvs)
sd_npv <- sd(npvs)
mean_accuracy <- mean(accuracies)
sd_accuracy <- sd(accuracies)
mean_auc <- mean(aucs)
sd_auc <- sd(aucs)

# Print the summary results
cat("Mean F1 Score:", mean_f1, "\n")
```

```
## Mean F1 Score: 0.9117161
```

```
cat("Standard Deviation of F1 Score:", sd_f1, "\n")
```

```
## Standard Deviation of F1 Score: 0.01688794
```

```
cat("Mean Sensitivity:", mean_sensitivity, "\n")
```

```
## Mean Sensitivity: 0.8488055
```

```
cat("Standard Deviation of Sensitivity:", sd_sensitivity, "\n")
```

```
## Standard Deviation of Sensitivity: 0.03237765
```

```
cat("Mean Specificity:", mean_specificity, "\n")
```

```
## Mean Specificity: 0.9861174
```

```
cat("Standard Deviation of Specificity:", sd_specificity, "\n")
```

```
## Standard Deviation of Specificity: 0.01248648
```

```
cat("Mean PPV:", mean_ppv, "\n")
```

```
## Mean PPV: 0.9856478
```

```
cat("Standard Deviation of PPV:", sd_ppv, "\n")
```

```
## Standard Deviation of PPV: 0.01008396
```

```
cat("Mean NPV:", mean_npv, "\n")
```

```
## Mean NPV: 0.8660385
```

```
cat("Standard Deviation of NPV:", sd_npv, "\n")
```

```
## Standard Deviation of NPV: 0.02509772
```

```
cat("Mean Accuracy:", mean_accuracy, "\n")
```

```
## Mean Accuracy: 0.9178571
```

```
cat("Standard Deviation of Accuracy:", sd_accuracy, "\n")
```

```
## Standard Deviation of Accuracy: 0.01397366
```

```
cat("Mean AUC:", mean_auc, "\n")
```

```
## Mean AUC: 0.9826695
```

```
cat("Standard Deviation of AUC:", sd_auc, "\n")
```

```
## Standard Deviation of AUC: 0.008463346
```

```
# Save the models, confusion matrices, and results table
save(models, file = "models.RData")
save(conf_matrices, file = "conf_matrices.RData")
save(results_table, file = "results_table.RData")
```

# **Testing Classifier 13- NN**

```
library(caret)
library(neuralnet)
library(pROC)
library(ROCR)

# Cargar el modelo desde el archivo
load("models.RData")

# Seleccionar el mejor modelo (por ejemplo, el primer modelo)
best_model <- models[[5]]

# Evaluar el modelo en un nuevo conjunto de prueba
new_test_data <- data.test
new_test_pred <- predict(best_model, new_test_data, type = "response")

# Extraer las probabilidades predichas
new_test_predicted_prob <- new_test_pred[, 1]
new_test_predicted_prob <- as.numeric(new_test_predicted_prob)

# Convertir probabilidades a predicciones binarias usando un umbral de 0.9
new_test_predicted_prob2 <- ifelse(new_test_predicted_prob > 0.95, 1, 0)
new_test_predicted_prob2 <- factor(new_test_predicted_prob2, levels = c(1, 0))
new_test_actual <- factor(new_test_data$perdida_bin, levels = c(1, 0))

# Calcular la matriz de confusión para el nuevo conjunto de prueba
new_test_confusion <- confusionMatrix(new_test_predicted_prob2, new_test_actual)
print(new_test_confusion)
```

```
## Confusion Matrix and Statistics
## 
##           Reference
## Prediction     1     0
##          1   176   182
##          0    41 23114
##                                           
##                Accuracy : 0.9905          
##                  95% CI : (0.9892, 0.9917)
##     No Information Rate : 0.9908          
##     P-Value [Acc > NIR] : 0.6745          
##                                           
##                   Kappa : 0.6077          
##                                           
##  Mcnemar's Test P-Value : <2e-16          
##                                           
##             Sensitivity : 0.811060        
##             Specificity : 0.992188        
##          Pos Pred Value : 0.491620        
##          Neg Pred Value : 0.998229        
##              Prevalence : 0.009229        
##          Detection Rate : 0.007485        
##    Detection Prevalence : 0.015226        
##       Balanced Accuracy : 0.901624        
##                                           
##        'Positive' Class : 1               
##
```

```
# Calcular el AUC
new_auc_value <- auc(new_test_actual, new_test_predicted_prob)
cat("AUC:", new_auc_value, "\n")
```

```
## AUC: 0.9829959
```

# **Confusion matrix Classifier 13**

```
#Confusion matrix 
draw_confusion_matrix(new_test_confusion)
```

# **Classifier 14- NN. Training and validation**

```
library(caret)
library(neuralnet)
library(data.table)  # Para crear la tabla comparativa

# Perform 10-fold cross-validation on the balanced training set
set.seed(69)
folds <- createFolds(data.train.balanced$perdida_bin, k = 10)
accuracies <- numeric(10)
f1_scores <- numeric(10)
sensitivities <- numeric(10)
specificities <- numeric(10)
ppvs <- numeric(10)  # Positive Predictive Values
npvs <- numeric(10)  # Negative Predictive Values
models <- list()  # Lista para almacenar los modelos de cada fold
conf_matrices <- list()  # Lista para almacenar las matrices de confusión de cada fold

for (i in 1:10) {
    # Segment your data by fold using the which() function 
    validationIndexes <- folds[[i]]
    validationData <- data.train.balanced[validationIndexes, ]
    trainData <- data.train.balanced[-validationIndexes, ]

    # Train the neural network model
   set.seed(69)
    n5 <- neuralnet(perdida_bin ~ nefrectomia+ perdida+ hemodialisi+ unidad+ terapia+ dialisi,
               data = trainData,
               hidden = c(2,2),
               linear.output = F,
               lifesign = 'full',
               threshold = 0.02,
               lifesign.step = 10,
               rep=1)

    
    # Store the model
    models[[i]] <- n5
    
    # Predict on the validation data
    predicted <- predict(n5, validationData, type = "response")
 
    # Convert probabilities to binary predictions using a threshold of 0.1
    predicted_prob2 <- ifelse(predicted[, 1] > 0.9, 1, 0)
    predicted_prob2 <- factor(predicted_prob2, levels = c(1, 0))
    validation.actual <- factor(validationData$perdida_bin, levels = c(1, 0))
    
    # Compute the confusion matrix and extract metrics
    conf_matrix <- confusionMatrix(predicted_prob2, validation.actual)
    conf_matrices[[i]] <- conf_matrix  # Store the confusion matrix
    accuracies[i] <- conf_matrix$overall['Accuracy']
    f1_scores[i] <- conf_matrix$byClass['F1']
    sensitivities[i] <- conf_matrix$byClass['Sensitivity']
    specificities[i] <- conf_matrix$byClass['Specificity']
    ppvs[i] <- conf_matrix$byClass['Pos Pred Value']
    npvs[i] <- conf_matrix$byClass['Neg Pred Value']
}

# Create a data table with the results
results_table <- data.table(
  Fold = 1:10,
  Accuracy = accuracies,
  F1_Score = f1_scores,
  Sensitivity = sensitivities,
  Specificity = specificities,
  PPV = ppvs,
  NPV = npvs
)

# Print the results table
print(results_table)
```

```
##      Fold  Accuracy  F1_Score Sensitivity Specificity       PPV       NPV
##     <int>     <num>     <num>       <num>       <num>     <num>     <num>
##  1:     1 0.9285714 0.9259259   0.8720930   0.9878049 0.9868421 0.8804348
##  2:     2 0.9166667 0.9113924   0.8470588   0.9879518 0.9863014 0.8631579
##  3:     3 0.8988095 0.9005848   0.8461538   0.9610390 0.9625000 0.8409091
##  4:     4 0.9226190 0.9312169   0.8979592   0.9571429 0.9670330 0.8701299
##  5:     5 0.9226190 0.9078014   0.8421053   0.9891304 0.9846154 0.8834951
##  6:     6 0.9285714 0.9154930   0.8552632   0.9891304 0.9848485 0.8921569
##  7:     7 0.9226190 0.9064748   0.8513514   0.9787234 0.9692308 0.8932039
##  8:     8 0.9285714 0.9250000   0.8705882   0.9879518 0.9866667 0.8817204
##  9:     9 0.9166667 0.9156627   0.8539326   0.9873418 0.9870130 0.8571429
## 10:    10 0.9345238 0.9325153   0.8837209   0.9878049 0.9870130 0.8901099
```

```
# Calculate the mean and standard deviation of the metrics
mean_f1 <- mean(f1_scores)
sd_f1 <- sd(f1_scores)
mean_sensitivity <- mean(sensitivities)
sd_sensitivity <- sd(sensitivities)
mean_specificity <- mean(specificities)
sd_specificity <- sd(specificities)
mean_ppv <- mean(ppvs)
sd_ppv <- sd(ppvs)
mean_npv <- mean(npvs)
sd_npv <- sd(npvs)
mean_accuracy <- mean(accuracies)
sd_accuracy <- sd(accuracies)

# Print the summary results
cat("Mean F1 Score:", mean_f1, "\n")
```

```
## Mean F1 Score: 0.9172067
```

```
cat("Standard Deviation of F1 Score:", sd_f1, "\n")
```

```
## Standard Deviation of F1 Score: 0.01098711
```

```
cat("Mean Sensitivity:", mean_sensitivity, "\n")
```

```
## Mean Sensitivity: 0.8620226
```

```
cat("Standard Deviation of Sensitivity:", sd_sensitivity, "\n")
```

```
## Standard Deviation of Sensitivity: 0.01836205
```

```
cat("Mean Specificity:", mean_specificity, "\n")
```

```
## Mean Specificity: 0.9814021
```

```
cat("Standard Deviation of Specificity:", sd_specificity, "\n")
```

```
## Standard Deviation of Specificity: 0.01216948
```

```
cat("Mean PPV:", mean_ppv, "\n")
```

```
## Mean PPV: 0.9802064
```

```
cat("Standard Deviation of PPV:", sd_ppv, "\n")
```

```
## Standard Deviation of PPV: 0.009798436
```

```
cat("Mean NPV:", mean_npv, "\n")
```

```
## Mean NPV: 0.8752461
```

```
cat("Standard Deviation of NPV:", sd_npv, "\n")
```

```
## Standard Deviation of NPV: 0.01714426
```

```
cat("Mean Accuracy:", mean_accuracy, "\n")
```

```
## Mean Accuracy: 0.9220238
```

```
cat("Standard Deviation of Accuracy:", sd_accuracy, "\n")
```

```
## Standard Deviation of Accuracy: 0.009900774
```

```
# Save the models, confusion matrices, and results table
save(models, file = "models.RData")
save(conf_matrices, file = "conf_matrices.RData")
save(results_table, file = "results_table.RData")
```

# **Testing Classifier 14- NN**

```
library(caret)
library(neuralnet)
library(pROC)
library(ROCR)

# Cargar el modelo desde el archivo
load("models.RData")

# Seleccionar el mejor modelo (por ejemplo, el primer modelo)
best_model <- models[[4]]

# Evaluar el modelo en un nuevo conjunto de prueba
new_test_data <- data.test
new_test_pred <- predict(best_model, new_test_data, type = "response")

# Extraer las probabilidades predichas
new_test_predicted_prob <- new_test_pred[, 1]
new_test_predicted_prob <- as.numeric(new_test_predicted_prob)

# Convertir probabilidades a predicciones binarias usando un umbral de 0.9
new_test_predicted_prob2 <- ifelse(new_test_predicted_prob > 0.95, 1, 0)
new_test_predicted_prob2 <- factor(new_test_predicted_prob2, levels = c(1, 0))
new_test_actual <- factor(new_test_data$perdida_bin, levels = c(1, 0))

# Calcular la matriz de confusión para el nuevo conjunto de prueba
new_test_confusion <- confusionMatrix(new_test_predicted_prob2, new_test_actual)
print(new_test_confusion)
```

```
## Confusion Matrix and Statistics
## 
##           Reference
## Prediction     1     0
##          1   175   297
##          0    42 22999
##                                          
##                Accuracy : 0.9856         
##                  95% CI : (0.984, 0.9871)
##     No Information Rate : 0.9908         
##     P-Value [Acc > NIR] : 1              
##                                          
##                   Kappa : 0.5017         
##                                          
##  Mcnemar's Test P-Value : <2e-16         
##                                          
##             Sensitivity : 0.806452       
##             Specificity : 0.987251       
##          Pos Pred Value : 0.370763       
##          Neg Pred Value : 0.998177       
##              Prevalence : 0.009229       
##          Detection Rate : 0.007443       
##    Detection Prevalence : 0.020074       
##       Balanced Accuracy : 0.896851       
##                                          
##        'Positive' Class : 1              
##
```

```
# Calcular el AUC
new_auc_value <- auc(new_test_actual, new_test_predicted_prob)
cat("AUC:", new_auc_value, "\n")
```

```
## AUC: 0.9827119
```

# **Confusion matrix Classifier 14**

```
#Confusion matrix 
draw_confusion_matrix(new_test_confusion)
```

# **Classifier 15- NN. Training and validation**

```
library(caret)
library(neuralnet)
library(data.table)  # Para crear la tabla comparativa

# Perform 10-fold cross-validation on the balanced training set
set.seed(69)
folds <- createFolds(data.train.balanced$perdida_bin, k = 10)
accuracies <- numeric(10)
f1_scores <- numeric(10)
sensitivities <- numeric(10)
specificities <- numeric(10)
ppvs <- numeric(10)  # Positive Predictive Values
npvs <- numeric(10)  # Negative Predictive Values
models <- list()  # Lista para almacenar los modelos de cada fold
conf_matrices <- list()  # Lista para almacenar las matrices de confusión de cada fold

for (i in 1:10) {
    # Segment your data by fold using the which() function 
    validationIndexes <- folds[[i]]
    validationData <- data.train.balanced[validationIndexes, ]
    trainData <- data.train.balanced[-validationIndexes, ]

    # Train the neural network model
   set.seed(69)
    n6 <- neuralnet(perdida_bin ~ nefrectomia+ perdida+ hemodialisi+ unidad+ terapia+ dialisi,
               data = trainData,
               hidden = c(2,2),
               linear.output = F,
               lifesign = 'full',
               threshold = 0.01,
               lifesign.step = 10,
               rep=1)

    
    # Store the model
    models[[i]] <- n6
    
    # Predict on the validation data
    predicted <- predict(n6, validationData, type = "response")
 
    # Convert probabilities to binary predictions using a threshold of 0.1
    predicted_prob2 <- ifelse(predicted[, 1] > 0.9, 1, 0)
    predicted_prob2 <- factor(predicted_prob2, levels = c(1, 0))
    validation.actual <- factor(validationData$perdida_bin, levels = c(1, 0))
    
    # Compute the confusion matrix and extract metrics
    conf_matrix <- confusionMatrix(predicted_prob2, validation.actual)
    conf_matrices[[i]] <- conf_matrix  # Store the confusion matrix
    accuracies[i] <- conf_matrix$overall['Accuracy']
    f1_scores[i] <- conf_matrix$byClass['F1']
    sensitivities[i] <- conf_matrix$byClass['Sensitivity']
    specificities[i] <- conf_matrix$byClass['Specificity']
    ppvs[i] <- conf_matrix$byClass['Pos Pred Value']
    npvs[i] <- conf_matrix$byClass['Neg Pred Value']
}

# Create a data table with the results
results_table <- data.table(
  Fold = 1:10,
  Accuracy = accuracies,
  F1_Score = f1_scores,
  Sensitivity = sensitivities,
  Specificity = specificities,
  PPV = ppvs,
  NPV = npvs
)

# Print the results table
print(results_table)
```

```
##      Fold  Accuracy  F1_Score Sensitivity Specificity       PPV       NPV
##     <int>     <num>     <num>       <num>       <num>     <num>     <num>
##  1:     1 0.9285714 0.9259259   0.8720930   0.9878049 0.9868421 0.8804348
##  2:     2 0.9166667 0.9113924   0.8470588   0.9879518 0.9863014 0.8631579
##  3:     3 0.8690476 0.8674699   0.7912088   0.9610390 0.9600000 0.7956989
##  4:     4 0.9226190 0.9312169   0.8979592   0.9571429 0.9670330 0.8701299
##  5:     5 0.9226190 0.9078014   0.8421053   0.9891304 0.9846154 0.8834951
##  6:     6 0.9345238 0.9230769   0.8684211   0.9891304 0.9850746 0.9009901
##  7:     7 0.9226190 0.9064748   0.8513514   0.9787234 0.9692308 0.8932039
##  8:     8 0.9166667 0.9113924   0.8470588   0.9879518 0.9863014 0.8631579
##  9:     9 0.9166667 0.9156627   0.8539326   0.9873418 0.9870130 0.8571429
## 10:    10 0.9345238 0.9325153   0.8837209   0.9878049 0.9870130 0.8901099
```

```
# Calculate the mean and standard deviation of the metrics
mean_f1 <- mean(f1_scores)
sd_f1 <- sd(f1_scores)
mean_sensitivity <- mean(sensitivities)
sd_sensitivity <- sd(sensitivities)
mean_specificity <- mean(specificities)
sd_specificity <- sd(specificities)
mean_ppv <- mean(ppvs)
sd_ppv <- sd(ppvs)
mean_npv <- mean(npvs)
sd_npv <- sd(npvs)
mean_accuracy <- mean(accuracies)
sd_accuracy <- sd(accuracies)

# Print the summary results
cat("Mean F1 Score:", mean_f1, "\n")
```

```
## Mean F1 Score: 0.9132929
```

```
cat("Standard Deviation of F1 Score:", sd_f1, "\n")
```

```
## Standard Deviation of F1 Score: 0.01866466
```

```
cat("Mean Sensitivity:", mean_sensitivity, "\n")
```

```
## Mean Sensitivity: 0.855491
```

```
cat("Standard Deviation of Sensitivity:", sd_sensitivity, "\n")
```

```
## Standard Deviation of Sensitivity: 0.02889913
```

```
cat("Mean Specificity:", mean_specificity, "\n")
```

```
## Mean Specificity: 0.9814021
```

```
cat("Standard Deviation of Specificity:", sd_specificity, "\n")
```

```
## Standard Deviation of Specificity: 0.01216948
```

```
cat("Mean PPV:", mean_ppv, "\n")
```

```
## Mean PPV: 0.9799425
```

```
cat("Standard Deviation of PPV:", sd_ppv, "\n")
```

```
## Standard Deviation of PPV: 0.01030498
```

```
cat("Mean NPV:", mean_npv, "\n")
```

```
## Mean NPV: 0.8697521
```

```
cat("Standard Deviation of NPV:", sd_npv, "\n")
```

```
## Standard Deviation of NPV: 0.02976427
```

```
cat("Mean Accuracy:", mean_accuracy, "\n")
```

```
## Mean Accuracy: 0.9184524
```

```
cat("Standard Deviation of Accuracy:", sd_accuracy, "\n")
```

```
## Standard Deviation of Accuracy: 0.01862333
```

```
# Save the models, confusion matrices, and results table
save(models, file = "models.RData")
save(conf_matrices, file = "conf_matrices.RData")
save(results_table, file = "results_table.RData")
```

# **Testing Classifier 14- NN**

```
library(caret)
library(neuralnet)
library(pROC)
library(ROCR)

# Cargar el modelo desde el archivo
load("models.RData")

# Seleccionar el mejor modelo (por ejemplo, el primer modelo)
best_model <- models[[4]]

# Evaluar el modelo en un nuevo conjunto de prueba
new_test_data <- data.test
new_test_pred <- predict(best_model, new_test_data, type = "response")

# Extraer las probabilidades predichas
new_test_predicted_prob <- new_test_pred[, 1]
new_test_predicted_prob <- as.numeric(new_test_predicted_prob)

# Convertir probabilidades a predicciones binarias usando un umbral de 0.9
new_test_predicted_prob2 <- ifelse(new_test_predicted_prob > 0.95, 1, 0)
new_test_predicted_prob2 <- factor(new_test_predicted_prob2, levels = c(1, 0))
new_test_actual <- factor(new_test_data$perdida_bin, levels = c(1, 0))

# Calcular la matriz de confusión para el nuevo conjunto de prueba
new_test_confusion <- confusionMatrix(new_test_predicted_prob2, new_test_actual)
print(new_test_confusion)
```

```
## Confusion Matrix and Statistics
## 
##           Reference
## Prediction     1     0
##          1   175   297
##          0    42 22999
##                                          
##                Accuracy : 0.9856         
##                  95% CI : (0.984, 0.9871)
##     No Information Rate : 0.9908         
##     P-Value [Acc > NIR] : 1              
##                                          
##                   Kappa : 0.5017         
##                                          
##  Mcnemar's Test P-Value : <2e-16         
##                                          
##             Sensitivity : 0.806452       
##             Specificity : 0.987251       
##          Pos Pred Value : 0.370763       
##          Neg Pred Value : 0.998177       
##              Prevalence : 0.009229       
##          Detection Rate : 0.007443       
##    Detection Prevalence : 0.020074       
##       Balanced Accuracy : 0.896851       
##                                          
##        'Positive' Class : 1              
##
```

```
# Calcular el AUC
new_auc_value <- auc(new_test_actual, new_test_predicted_prob)
cat("AUC:", new_auc_value, "\n")
```

```
## AUC: 0.9826611
```

# **Confusion matrix Classifier 15**

```
#Confusion matrix 
draw_confusion_matrix(new_test_confusion)
```
