# Supplementary material for: Using Natural Language Processing and Machine Learning to classify the status of kidney allograft in Electronic Medical Records written in Spanish
Source: PLoS One. 2025 May 8;20(5):e0322587. doi: 10.1371/journal.pone.0322587 (PMC12061128; doi:10.1371/journal.pone.0322587)
Supplement: Attachment — Submitted filename: Response to Reviewers.docx [file pone.0322587.s005.docx]

**'Response to Reviewers'**

**Journal requirements**

**If applicable, we recommend that you deposit your laboratory protocols in protocols.io to enhance the reproducibility of your results.**

*R/ Not applicable, our protocol does not pertain to laboratory procedures.*

**Please ensure that your manuscript meets PLOS ONE's style requirements, including those for file naming. The PLOS ONE style templates can be found at 
https://journals.plos.org/plosone/s/file?id=wjVg/PLOSOne_formatting_sample_main_body.pdf and 
https://journals.plos.org/plosone/s/file?id=ba62/PLOSOne_formatting_sample_title_authors_affiliations.pdf**

*R/ We have thoroughly reviewed our manuscript and confirm that it adheres to all of PLOS ONE’s style requirements.*

**Please note that PLOS ONE has specific guidelines on code sharing for submissions in which author-generated code underpins the findings in the manuscript. In these cases, we expect all author-generated code to be made available without restrictions upon publication of the work. Please review our guidelines at https://journals.plos.org/plosone/s/materials-and-software-sharing#loc-sharing-code and ensure that your code is shared in a way that follows best practice and facilitates reproducibility and reuse.**

*R/ To comply with these guidelines, we shared the code in a public repository. This will allow other researchers to access, reproduce, and build upon our work, thereby enhancing the transparency and impact of our research.*

*Code for models and validation can be found at:*

<https://www.kaggle.com/code/aegarcialo/data-balanced-logistic-regression-and-random-fore>

*It is also attached in Supplemental material (S3 and S4)*

**We note that you have indicated that there are restrictions to data sharing for this study. PLOS only allows data to be available upon request if there are legal or ethical restrictions on sharing data publicly. For more information on unacceptable data access restrictions, please see** [**http://journals.plos.org/plosone/s/data-availability#loc-unacceptable-data-access-restrictions**](http://journals.plos.org/plosone/s/data-availability#loc-unacceptable-data-access-restrictions)

*R/ We acknowledge your concern regarding data sharing. The structured data after preprocessing from this study is deposited in a public repository.*

*It can be found at:*

[*https://www.kaggle.com/ds/6032764*](https://www.kaggle.com/ds/6032764)

*DOI: 10.34740/KAGGLE/DS/6032764*

*The original clinical data with unstructured text will be available upon request to the main author via email*

**We require you to either (1) present written permission from the copyright holder to publish these figures specifically under the CC BY 4.0 license, or (2) remove the figures from your submission:**

*R/ We acknowledge your request regarding the figures. The image presented in the previous version of the paper was our own design, but it was created using a free version of a program, which resulted in watermarks. We have now replaced Figure 1 with a new version (created by the authors), ensuring that the design was created and downloaded using the Pro version of the program (Pro content license). This allows us to use and publish the elements without any watermarks.*

*The image was designed (by the authors) using Canva Pro, which grants non-exclusive licenses to use content under the* ***Pro Content License (also known as One Design Use License)****. This allows us to use and publish the elements of the design in accordance with the license terms, ensuring that all components of the design comply with usage requirements.*

*More information can be found here: https://www.canva.com/policies/content-license-agreement/*

**Comments to the Author**

Review Comments to the Author

Reviewer #1: The article reports a method for identifying graft loss in electronic medical records. It highlights the creation of a database of 2712 patients with a total of 117566 unstructured medical records. A text feature extraction scheme based on NLP by means of word and N-gram identification and the use of machine learning models for a binary classification scheme is reported.

Although there is an interesting contribution in the creation of the database and the application in medical records related to kidney transplantation, there are several methodological problems in the study, and the contribution from the technique is not clear.

**The major findings are described below:**

Comment:
**- The references cited in the introduction regarding the use of NLP in the identification of medical conditions are not state of the art. The most current one is from 2022 and does not refer to the use of large language models, which is what is currently used.**

*R/ Thank you for your feedback. We have updated the references in the introduction to include the most recent studies, particularly those focusing on the use of large language models in the identification of medical conditions.*

*We also added the following text in the introduction: “Conversely, recent advancements in large language models (LLMs) have significantly transformed the landscape of NLP and artificial intelligence (AI). While generative LLMs have been widely explored for healthcare data analysis, their application in real-world EMR data remains limited due to significant privacy concerns and current challenges such as bias, hallucinations, and impersonal responses (10,11)”.*

Comment:

**-The methods used for word count and N-grams do not take context into account. For example, it does not take into account whether a term is present, absent or a possibility. This changes the interpretation and can affect the result. On the other hand, the methodology used may depend on whether the terms have typographical errors (which is common in medical records).** **Although a method of spelling correction is reported, it is not optimal.**

*R/. We acknowledge that the methods used for word count and N-grams do not fully account for context, which can indeed influence the interpretation and results. On the other hand, we have conducted a thorough spell check and analyzed the context word by word to address these issues. We added the following text in the methods section: “Following the removal of stopwords, medical abbreviations were expanded into full words, as detailed in S1 Table A.4. A comprehensive spell-checking process was implemented using an automated spell check tool, which was supplemented by expert supervision (AG-L) to identify and correct errors in context. The expert meticulously reviewed all words to ensure accuracy. The list of original and corrected words can be found in Supplementary File (S2).*

*This rigorous process revealed various types of spelling errors, which were categorized into accentuation errors, omission errors, substitution errors, and others (Error definition listed in S1 Table A.5). All identified errors were corrected within the text, and in cases where the words had no meaningful context (less than 0.1%), they were replaced with placeholders”.*

*We believe that with these corrections and the detailed context analysis, it is possible to bypass the need for analyzing whether terms are present, absent, or possibilities. By ensuring the accuracy and context of each word, we have mitigated the potential misinterpretations that could arise from typographical errors or lack of meaning in context.*

Comment

**-It is not clear whether the results presented are from a test set. In Table 3, if the results are from cross-validation they should be presented with standard deviation. -When reviewing the confusion matrix, it appears that all medical records were used.**

*R/ Thank you for your observation. In our previous analysis, some results were indeed analyzed using out-of-bag (OOB) estimates of some Random Forest models. However, we have implemented sample balancing and re-evaluated all models using the test set for evaluation.*

*We have updated Table 3 to reflect these changes, ensuring that all results are now derived from the balanced test set. Additionally, for results obtained through cross-validation, we have included the standard deviation of the measure of overall accuracy to provide a clearer understanding of the variability in our overall performance metric.*

Comment

**-On the other hand, it is observed that the high specificity and high accuracy reported is an effect of class imbalance. This is verified when it is seen that the number of false positives is similar to the true positives, giving an accuracy of 0.519.**

*R/We acknowledge that the high specificity and accuracy initially reported were influenced by class imbalance. To address this, we have implemented sample balancing using the under-sampling technique on the registries without graft loss, which constituted the majority, and re-evaluated all models with the balanced sample.*

*The updated results, derived from the balanced dataset, provide a more accurate representation of model performance. Table 3 reflect these changes and the code and output is provided in supplemental material S3 and S4.*

*It is important to notice that even in this balanced dataset all our models present relatively high performance on all metrics, with values of accuracy and F1 scores above 0.93.*

*We appreciate your feedback and believe these adjustments were conducive to a more rigorous evaluation of model performance, and thus enhance the validity and reliability of our findings.*

Comment

**-The most important measure for the analysis should be the F1 score, which is reported to be 0.651 (Figure 4.a).**

**R/** *We agree that the F1 score is a crucial measure for evaluating model performance. In response to your comment, we have included the F1 score as one of the key metrics in the analysis of all models. After balancing the sample, the F1 score improved substantially, and now ranges between 0.935 and 0.955*

Comment

**-When reviewing the confusion matrices of the supplementary material, it is not understood why for some models the matrix is reported with 1063 positive cases or other models with 101 positive cases and others with 119.**

*R/ Thank you for your observation. In our previous analysis, some results were indeed derived using out-of-bag (OOB) samples, which led to discrepancies in the confusion matrices, such as the varying number of positive cases reported.*

*To address this issue, we have re-evaluated all models using a balanced sample. All reported results are now based on the balanced test set using cross-validation. This ensures consistency and accuracy in the confusion matrices and other performance metrics.*

Other suggestions:

**The manual labels were made by a single specialist. At least two specialists are suggested to perform concordance measurements.**

*R/ Thank you for your comment. Due to practical constraints, it was not feasible to have the review conducted by two specialists. However, to ensure the correct classification of the clinical records, we developed a list of predefined criteria corresponding to graft loss. These criteria were meticulously established in consultation with clinical experts and followed strictly by the reviewer to ensure the accuracy and reliability of the classification process.*

*We have added the following clarification in the manuscript:*

*“These criteria were meticulously established in consultation with clinical experts to ensure accuracy and reliability in the classification process. This collaborative approach with clinical experts provided a robust framework for the manual review, enhancing the validity of the findings”.*
