# Supplementary material for: Using Natural Language Processing and Machine Learning to classify the status of kidney allograft in Electronic Medical Records written in Spanish
Source: PLoS One. 2025 May 8;20(5):e0322587. doi: 10.1371/journal.pone.0322587 (PMC12061128; doi:10.1371/journal.pone.0322587)
Supplement: Attachment — Submitted filename: Response_to_reviewers_auresp_2.docx [file pone.0322587.s006.docx]

**'Response to Reviewers'**

**Comments to the Author**

Review Comments to the Author

**Reviewer #1:**

**The authors improved the manuscript according to the suggestions in the first review. However, the following major issues have been addressed:**

**In the methods section, you explain how you balanced the dataset. That is a necessary step in training the models. However, testing the models in the balanced dataset differs from a real scenario. Please train in a balanced dataset and test in an unbalanced test set. If you cannot test in an unbalanced scenario, you can describe this fact as a limitation of the study.**

*R/ Thank you for your valuable comments. We have accepted your suggestion and made the requested changes. The model has now been trained on a balanced dataset and tested on an unbalanced test set. The results of these tests have been included in the Results section of the revised manuscript (Table 3). Additionally, we have updated the Methods section to explain this process in detail.*

**When you work with machine learning models, splitting the dataset into a train set and a test set is necessary. You can perform the cross-validation in the training set, but you must also report the performance metrics in the test set. It is better if the test set is unbalanced, although the train set is balanced. You must declare this in the limitations if you can follow these steps.**

*R/ Thank you for your insightful comments. We have accepted your suggestion and made the requested changes. The following modifications were implemented:*

1. *The dataset was initially partitioned using an 80/20 split, where 80% of the data was allocated for training and validation, and the remaining 20% was reserved for testing.*
2. *The training and validation set underwent a 10-fold cross-validation process. In this method, the data was divided into 10 equal folds, with 9 folds used for training and 1 fold used for validation in each iteration. This cross-validation process was repeated 10 times, ensuring that each fold was used as the validation set exactly once.*
3. *The final model performance was evaluated on the test set, which comprised the remaining 20% of the data.*

*These changes have been detailed in the Methods section, and the performance metrics have been reported in the Results section. As suggested, the test set was unbalanced to better reflect real-world scenarios.*

**Additionally, it is necessary to declare the hyperparameters used in the machine learning models. If you perform a hyperparameter optimization, you must state the methods used.**

*R/ Thank you for your valuable comments. We attempted to use hyperparameter optimization; however, it required significant computational resources, which we do not have. As a result, each model reported has the best combination of hyperparameters tested manually. We have declared the in the Methods section that hyperparameter optimization techniques were not used and, in the limitations section, we declared* ***“Furthermore, hyperparameter optimization techniques, which could significantly enhance model performance, were not utilized due to the high computational demands, making it unfeasible for this particular study.”***

*We appreciate your observation, as it has helped improve the clarity and transparency of our study.*

**In the key findings section, avoid using the word "demonstrated." This preliminary study needs other validations to be "demonstrated." Instead, you can use words like "show," "suggest," and others.**

*R/ Thank you for your feedback. We have made the suggested changes in the key findings section. The word "demonstrated" has been replaced.*

Reviewer #2:

General comments

**In this paper, the authors developed several classification models using features extracted from unstructured clinical texts to predict the kidney allograft. The authors did a lot of manual work to implement their models. However, there are some limitations in the current manuscript.**

Major comments

**1.The innovation of methodology seems weak. The authors employed common methods to extract features from clinical text, select features, and develop prediction models.**

*Thank you for your feedback. You are correct that the methods we used, such as information extraction through NLP, variable selection with LASSO regression, and the construction of prediction models, are well-established and widely supported in the scientific literature. However, the contribution of this study lies in applying these widely used techniques to address a persistent problem: the management of abundant unstructured clinical data and the manual work it entails. Additionally, this study makes a significant contribution to the field of transplants, which lacks published research on this topic, and it is the first study in the field working with text written in Spanish.*

*Furthermore, we recognize that this is a first step, and future work could leverage Large Language Models (LLMs) and other developments in artificial intelligence to further enhance the analysis and classification capabilities.*

**2.If the “Text mining search” procedure leads to some false negative samples? Some positive samples’ clinical text may not contain the listed keywords, which will be neglected by the following manual review.**

*R/ It is possible that the text mining search did not include some keywords. However, we made every effort to minimize this risk. The search criteria were clinically reviewed, and upon examining the texts, many patients with graft loss contain more than one indicative word in their texts. Therefore, we expect that at this stage the loss of cases will be minimal.*

**3.I noticed that some medical abbreviations like “k” and “h” in Table A.4 are the same as the custom stopwords. As the authors first removed the custom stopwords and then expanded the medical abbreviations, how to ensure that the medical abbreviations are not deleted by mistake.**

*R/Thank you for noticing this. In the Methods section, we clarified that common stopwords were removed before expanding medical abbreviations. Additionally, custom stopwords were removed at the end of the preprocessing phase, ensuring that medical abbreviations were not mistakenly deleted.*

**4.Please indicate how the feature selection was conducted. The current study did not specify whether the feature selection was performed on the entire dataset or on the training set. Conducting on the entire dataset may lead to test data leakage.**

*R/ Thank you for your observation. Feature selection was conducted on the training set, as specified in the Methods section. We used two techniques: bivariate analysis with a p-value threshold of ≤ 0.01 for a statistically significant association with graft loss, and LASSO (least absolute shrinkage and selection operator) to select the highest coefficients.*

**5.The authors should specify how they split training, validation, and test sets when using 10-fold cross validation. In my opinion, the current study just selected several groups of hyperparameters first. Then, the authors trained the models with these hyperparameters and tested the models using a 10-fold CV. If it is true, the current results are just validation results but not test results, which can not reflect the true generalization ability of the models. The authors should use the 10-fold CV first. During each fold iteration, the authors should split some samples from the 9-fold data as the validation set and leave the remaining samples of the 9-fold data as the training set. After that, they should conduct feature selection on the training set, train the models using the selected features and different hyperparameters, validate the trained models on the validation set to select the best hyperparameters, and finally, test the selected models on the test set.**

*R/ Thank you for your insightful comments. We have accepted your suggestion and made the requested changes. The following modifications were implemented:*

1. *The dataset was initially partitioned using an 80/20 split, where 80% of the data was allocated for training and validation, and the remaining 20% was reserved for testing.*
2. *The training and validation set underwent a 10-fold cross-validation process. In this method, the data was divided into 10 equal folds, with 9 folds used for training and 1 fold used for validation in each iteration. This cross-validation process was repeated 10 times, ensuring that each fold was used as the validation set exactly once.*
3. *Feature selection was performed in the training set.*
4. *The final model performance was evaluated on the test set, which comprised the remaining 20% of the data.*

*These changes have been detailed in the Methods section, and the performance metrics have been reported in the Results section. As suggested, the test set was unbalanced to better reflect real-world scenarios.*

**6.The current study did not compare the developed models with SOTA models, such as BERT or ChatGPT. Besides, they should also compare with the Rule-based method.**

*R/ Thank you for your valuable feedback. We acknowledge that the current study did not compare the developed models with state-of-the-art (SOTA) models such as BERT or ChatGPT. Future work will include these comparisons to provide a more comprehensive evaluation.*

*In the manuscript, we recognized that the use of large language models would add value and will be considered for future work. Conducting these comparisons may be beyond the scope of this study, given that it was initiated in 2023 when the use of these models was still emerging. Nonetheless, we believe that our study contributes valuable insights into the use of natural language processing tools and serves as a first step towards more complex models.*

**The results in Table 3 are validation results with different hyperparameters, which is inappropriate.**

*R/ Thank you for your observation. We have made the suggested changes by training the models on 9-folds of the cross-validation, validated on 1-fold with different hyperparameters, and tested on the imbalanced test set to evaluate the model in a real-world scenario. We selected the top three algorithms from each class—Logistic Regression, Random Forest, and Neural Network—for comparison. As a result, the outcomes in Table 3 have been updated accordingly.*

Reviewer #3: (No Response)
