# Supplementary material for: Using Natural Language Processing and Machine Learning to classify the status of kidney allograft in Electronic Medical Records written in Spanish
Source: PLoS One. 2025 May 8;20(5):e0322587. doi: 10.1371/journal.pone.0322587 (PMC12061128; doi:10.1371/journal.pone.0322587)
Supplement: Attachment — Submitted filename: Response_to_reviewers_auresp_3.docx [file pone.0322587.s007.docx]

**Journal Requirements:**
 **Please review your reference list to ensure that it is complete and correct. If you have cited papers that have been retracted, please include the rationale for doing so in the manuscript text, or remove these references and replace them with relevant current references. Any changes to the reference list should be mentioned in the rebuttal letter that accompanies your revised manuscript. If you need to cite a retracted article, indicate the article’s retracted status in the References list and also include a citation and full reference for the retraction notice.**

*R/ We have carefully reviewed our reference list and confirm that it is now complete and correct. Additionally, we have verified each cited article and confirm that none have been retracted.*

**Reviewers' comments:**

Reviewer #1: The authors have addressed the suggestions in the previous review, incorporating the proposed revisions. The modifications significantly improve the paper's clarity and coherence.

**Some minor issues:

1. Please include the link to the dataset repository in the "Data source and study population" section to make it available to readers.**

*R/We have included the reference to the dataset repository in the "Data Source and Study Population" section, and it has been properly cited.*

*Thank you for your suggestion.*

**2. In the "Comparison with other models" section, the authors claim: "When comparing our NLP algorithms to other state-of-the-art techniques in similar domains, our models showed considerable advantages in terms of discrimination, calibration, accuracy, and sensitivity." And, in key findings the authors wrote: "the Random Forest model outperformed the others achieving an AUC of 0.98 and an F1 score of 0.65, indicating strong discrimination between EMRs that recorded graft loss and those that did not, as well as a reasonable balance between precision and recall." However, discussing the implication of the low PPV (0.56) and the modest sensitivity is necessary. Although the results reach high specificity (0.99) and moderate F1 score (0.65), the PPV of 0.56 tells us that there are still many predicted positives that actually are not. In highly imbalanced problems, the PPV often suffers.**

*R/ We appreciate your feedback. In response to your comment, we have made the necessary clarification in the discussion section. We have added the following statement:*

- *Key findings:*

*"Notably, the model showed a low PPV (0.56) and modest sensitivity (0.76), indicating that a significant number of predicted positives are false positives, which implies additional manual work to verify these cases."*

- *Comparison with other models:*

*“However, while these models can be useful in clinical practice, manual work may still be required due to the high number of false positives”.*

*This addition addresses the implications of the low PPV and modest sensitivity, highlighting the need for further manual verification in practical applications*

**3. The abstract's result section focuses mainly on AUC results in the validation dataset. It is more important to discuss the results in the test set. Consider that a high ROC AUC and accuracy in highly imbalanced datasets can be misleading because the majority class can strongly influence the metric. Then, it is better to show the F1-score, sensitivity, and specificity results. This recommendation is an important fact in the discussion section, too.**

*R/ We have adjusted the abstract in the results and conclusion sections following your suggestion. The abstract now focuses on the results from the test set, emphasizing the importance of the F1-score, sensitivity, and specificity rather than just the AUC.*

*The discussion section also included this recommendation.*

**Reviewer #2: Thanks for considering my comments. For my fifth comment, the authors conducted their experiment in a different way, but it's OK. One more thing I need to verify is that the authors performed the 10-fold cross-validation on the training-validation set to make the hyperparameter selection, then re-train the model using the selected hyperparameters on the entire training-validation set as the final model and finally tested the final model on the test set?**

*R/ Thank you for your comment. Indeed, we first selected the hyperparameters using the training-validation set and trained the models with these chosen hyperparameters on the same training-validation set. Based on the results of the 10-fold cross-validation, we then tested the models on the test set. The final selected and tested hyperparameters for both validation and test sets are detailed in the supplementary code file, where we retained the best-performing hyperparameters.*

Reviewer #3: (No Response)
